# Why Do You Ride?: A Characterization of Mountain Bikers, Their Engagement Methods, and Perceived Links to Mental Health and Well-Being

**DOI:** 10.3389/fpsyg.2018.01642

**Published:** 2018-09-19

**Authors:** Lisa Roberts, Gareth Jones, Rob Brooks

**Affiliations:** School of Clinical and Applied Sciences, Leeds Beckett University, Leeds, United Kingdom

**Keywords:** mountain biking, mental health, extreme sports, outdoor activities, nature, health promotion, well-being

## Abstract

Mountain biking is an increasingly popular outdoor activity on the extreme sport continuum. Extreme and high-risk sports have been investigated using a variety of motivational theories with sensation seeking a dominant theme; however, behavioral and motivational homogeneity within these types of populations should not be assumed. Recent studies have highlighted the therapeutic potential of extreme sports and similar outdoor activities. The aim of this study was to describe the characteristics of mountain biking participants, their engagement methods, and perceived benefits to mental health and well-being. This was a cross-sectional survey and participants were recruited via social media. An online questionnaire specific to the domain of mountain biking was developed. Analysis of the full sample (*n* = 1,484) and of three independent paired sub-samples was conducted using SPSS. The sub-samples compared the results of males and females; younger and older riders; and those who have recently engaged in downhill mountain biking and those who have not. The results have succeeded in identifying some disparities in mountain biker characteristics and engagement methods. The results suggest that some riders found pleasure in higher risk engagement. The study proposes various explanations for the disproportion of women in mountain biking. Irrespective of the confounding factors related to rider characteristics or engagement methods, mountain bikers reported copious benefits to mental health and well-being related to their engagement. There was a high reported usage of mountain biking as a coping strategy. As such, this study provides insights that could inform the development of outdoor activities as interventions for mental health.

## Introduction

Extreme sports are described as activities where a mismanaged mistake or accident would most likely result in death (Brymer, 2005). However, activities that bear little resemblance to one another, requiring different levels of commitment and skill (such as rope-free climbing and bungee jumping), have been described as *extreme sports* (Brymer, 2010). Single activities are also regularly deemed to be extreme across the board, irrespective of the myriad possible ways to participate. A primary consequence is that variability in motivational orientation of participants of extreme sports, and participants' methods of engagement, have largely been overlooked (Jones et al., 2015; Clough et al., 2016; Zajc and Berzelak, 2016).

Mountain biking is a modern activity consisting of various off-road cycling disciplines. Participation varies from cycling on bridleways and canal towpaths, to more hazardous and challenging riding on extreme terrain. The International Mountain Biking Association (IMBA) estimated that the yearly participation figures for mountain biking in the United Kingdom was ~5 million in 2005 (Corporate Research Associates, 2010, p. 41); having grown considerably in popularity over the last 30 years (Moularde and Weaver, 2016; Poulson, 2016). Mountain biking has been given an assortment of labels; for example: a lifestyle sport (McCormack, 2017); a serious-leisure activity “on the hard-soft adventure continuum” (Taylor, 2010, p. 270); and an action and adventure sport (Immonen et al., 2017). Downhill mountain biking has been defined as an extreme sport (Becker et al., 2013) and an extreme sport subculture (Hagen, 2013). Becker and Moroder (2017) argue that all forms of off-road biking done in mountainous regions should be considered “extreme” (p. 149). Recent evidence regarding the morbidity associated with mountain biking suggested that it carries a “significant risk of life-threatening injury across all levels of participation” (Jeys et al., 2001, p. 197). Within this study mountain biking is considered *an activity on the extreme sports continuum*.

Historically, psychological research into extreme and adventure sports has largely been concerned with sensation-seeking, impulsivity and risk-taking behavior (Brymer and Schweitzer, 2017). The emphasis on risk has led to participation in extreme sports often being understood, until recently, as little more than thrill-seeking, deviant behavior (Zuckerman, 2000) carried out by “adrenaline-junkies.” The popular mountain bike media has also tended to over-emphasize the danger perceived in the sport, often portraying an ideology of hegemonic masculinity (Poulson, 2016). A survey found that 60.6% (*n* = 436) of females believed that the perception of mountain biking as “hard-core” was deterring women from participating (International Mountain Biking Association, 2010).

Recent research is more aligned to the lived experience of participants. Participation in high-risk activities does not necessarily equate to overestimation of abilities, or impulsive, irresponsible behavior (Llewellyn et al., 2008; Brymer, 2010). Rather, it is argued that hazards are usually mediated by the participant through a process of building competence and skill through experience; as opposed to taking risks for risk's sake (Llewellyn and Sanchez, 2008; Taylor, 2010; Lynch and Dibben, 2016; Frühauf et al., 2017). Participants may still experience fear as they test their mental and physical capabilities in challenging situations (Dodson, 1996; Lyng, 2005); but the fear could be understood as a sign that one is “pushing up against limitations and breaking throughboundaries” (Willig, 2008, p. 696), as opposed to recklessness. Contemporary literature has also included studies on the meaningfulness of extreme sports and links to psychological well-being (e.g., Willig, 2008; Brymer and Oades, 2009; Taylor, 2010; Cycling UK, 2017; Immonen et al., 2017). Evidence is also building to suggest that mainstream interventions for mental health should include extreme and adventure sports (Clough et al., 2016).

Recent mountain biking research also challenges the traditional perspective. Findings propose that regular off-road riders “generally do not fit the stereotype of mountain bikers as adrenaline-junkies” (Cycling UK, 2017, p. 7). Thrill-seeking should not be ignored - indeed, mountain biking participants have indicated that speed and adrenaline can be a motivation for participation (International Mountain Biking Association, 2010). There is also evidence to suggest that thrill-seeking may play a bigger part in initial motivation to engage in such activities, but that a desire to master skills and maintain health and well-being soon becomes more dominant (Willig, 2008; Cycling UK, 2017). Evidence is building to suggest that mountain biking participants are also motivated by intense positive emotions and fulfilling experiences (Dodson, 1996); challenge; opportunities for self-responsibility; development of identity; the aesthetics of the natural environment (Lynch and Dibben, 2016; Moularde and Weaver, 2016).

Mental health problems are a growing public health concern. Depression is now the leading cause of disability and ill health worldwide, increasing the risk of substance misuse and suicide (World Health Organization, 2018). The evidence supporting positive physical and psychosocial health outcomes in relation to nature-based activities and experiences is substantial (Brymer et al., 2010; Ryan et al., 2010; Scheinfeld et al., 2011; Mitchell, 2013; Martyn and Brymer, 2016; Yeh et al., 2016; Lawton et al., 2017). The natural environment is an integral part of the mountain biking experience, offering participants a unique way to connect with nature (Siderelis et al., 2010), and acts as an important motivational factor (Taylor, 2010; Davidson and Stebbins, 2011; Lynch and Dibben, 2016). Despite this, mainstream health interventions rarely include outdoor adventure activities or extreme sports (Clough et al., 2016).

In summary, recent evidence suggests behavioral and motivational homogeneity within these types of populations should not be assumed; and the various ways of participating in mountain biking appear to belong on several points along the extreme sport continuum. Thrill-seeking may motivate some participants to engage in mountain biking. The natural environments in which these activities take place are thought to be both an important motivational factor as well as a catalyst for improved well-being. Evidence is growing regarding the potential for extreme sports to contribute to positive mental health; however, there is a shortage of evidence concerning mountain biking. The aim of this study therefore was to describe the characteristics of mountain bikers, their engagement methods, and perceived benefits to psychological well-being.

## Methods

### Participants and procedure

This was a retrospective cross-sectional study utilizing an online survey to gather quantitative data on demographics, riding habits, preferences and motivations of a self-selecting sample of international mountain bikers. The only inclusion criterion was being a mountain biker aged 16 or over. Ethical approval was granted by Leeds Beckett University prior to the dissemination of the survey in June 2016. Informed consent was gained from each participant by ticking a check box before being able to continue with the survey. The survey comprised 68 separate items and was presented over 7 pages. A snowball sampling method was utilized (Bryman, 2016) by means of social media (Facebook and Twitter), and the survey remained live for three weeks.

### Measure

A standardized measure of the characteristics of mountain bikers and their engagement was not available. Quantitative studies on other extreme or adventure sports have usually used or adapted measures developed to explore personality traits or other psychological constructs (e.g., Llewellyn et al., 2008; Skår et al., 2008; Castanier et al., 2010; Hill et al., 2017). Due to this incongruity in current measurement, a survey tool was developed to provide an activity-specific approach to understanding the characteristics of the mountain biking population and constructs behind participation at a less traditional level of analysis (see Supplementary Material).

### Methodology and construct development

A sequential exploratory strategy (Cresswell, 2009) was mirrored in the study methodology. Step one: a rigorous synthesis of existing literature (prior to April 2016) on action or adventure activities (both qualitative and quantitative) facilitated the latent construct development of the survey. Step two: quantitative data collection, followed by analysis and interpretation. The construct development of the survey followed three specific stages as identified by Bearman and Dawson (2013): (1) key themes were identified in existing literature, (2) any inconsistencies or contradictions were acknowledged, (3) themes were ordered into over-arching categories that became themed sections of the survey. Steps were taken to address the potential for bias stemming from the subjective interpretation of context-dependent findings characteristic of qualitative synthesis; for example, emergent themes thought to be irrelevant to mountain biking were nonetheless included. These sections of the survey comprised statements with five Likert response options (Likert et al., 1993) ranging from “Strongly Agree” to “Strongly Disagree,” with a mid-point of “Neutral.” An additional section of the survey captured nominal data such as demographics and riding frequency of participants. Three mountain bikers piloted the survey. Minor adjustments were made included the re-wording of some questions and the reversal of some scales; and a small number of additional questions were added.

### Data analysis

Our study presents selected data from the larger survey. The “Agree” and “Strongly Agree” responses to Likert scale questions were merged, as were the “Disagree” and “Strongly Disagree” responses, to create a clear picture of participant attitude and to mitigate against central tendency bias. Responses were coded and data was analyzed descriptively using SPSS version 24 (SPSS Inc, Chicago, USA). To facilitate a deeper investigation, the sample was then split into three sub-sets of dichotomous variables. These were: (1) those who had ridden downhill in the past 2 years (*downhill riders*) and those who had not (*non-downhill riders*); (2) males and females; and (3) those aged 35 or under (*younger riders*), and those aged 36 or over (*older riders*). These categories were decided on for the following reasons: (1) comparing downhill riders with non-downhill riders may highlight differences between participants who engage in what is arguably the most high-risk form of mountain biking, compared with those who do not. (2) The literature has consistently shown that males dominate this activity (e.g., Cycling UK, 2017), therefore analyzing differences between males and females may provide some insight. (3) Previous literature has argued that as participants age, their motivations may change (e.g., Willig, 2008), therefore analyzing how age influences the results may facilitate a description of these differences. These three sub-samples were all measured using clear, unambiguous categorical data. The Likert items were treated as ordinal data, and conclusions drawn from these single items have been treated cautiously (Gilem and Gilem, 2003), and for the most part only in conjunction with other Likert items or alongside the nominal data results.

The use of parametric or non-parametric tests when analyzing Likert data is much debated (Sullivan and Artino, 2013). Certain researchers claim that both methods hold comparable power if the sample size is large and meets the required assumptions; others argue that using parametric methods can never be justified (De Winter and Dodou, 2010). As Likert data is ordinal, non-parametric testing is arguably the more appropriate method of analysis. The Mann–Whitney *U*-test was used in this study to compare the responses within the independent samples (male/female; downhill/non-downhill; 35 years or under/36 years or over). Cohen's classification has been used as a standardized measure to interpret effect size. Mann–Whitney results are reported in the main body of this paper only if *p* ≤ 0.001. See Tables 1–3 for all Mann–Whitney test results.

**Table 1:** Results from Mann–Whitney *U*-tests comparing male and female rider responses.

	**Mann–Whitney *U***	***Z***	**Asymp. Sig. (2-tailed)**	***n***	***r***
I participated in MTB as a child	95,719	−6.56	0.000	1,445	−0.17
Level	98,696	−5.84	0.000	1,448	−0.15
Age	126,372	−0.14	0.889	1,444	0.00
Length of time participating	101,654	−4.96	0.000	1,448	−0.13
Frequency of participation (winter)	115,815	−2.18	0.029	1,444	−0.06
Frequency of participation (summer)	117,036	−1.25	0.210	1,419	−0.03
Perceived risk in personal participation	120,333	−1.45	0.148	1,447	−0.04
Preference (group or alone)	123,487	−0.79	0.427	1,449	−0.02
I participate in MTB for the adrenaline rush	118,594	−2.34	0.019	1,449	−0.06
MTB fulfills my need for adventure	124,719	−0.73	0.465	1,447	−0.02
I enjoy the risk inherent in MTB	97,844	−6.37	0.000	1,448	−0.17
I am not a risk taker	120,684	−1.23	0.219	1,443	−0.03
I like appearing adventurous to others	120,734	−1.28	0.199	1,444	−0.03
MTB encourages me to explore my local countryside	124,882	−1.05	0.294	1,447	−0.03
I love being outdoors	126,075	−1.47	0.141	1,448	−0.04
MTB allows me to feel more connected to nature and the world around me	118,735	−2.19	0.029	1,442	−0.06
Being outdoors helps me to de-stress	126,183	−1.05	0.291	1,447	−0.03
If the weather in bad, I don't go riding	121,918	−0.99	0.324	1,445	−0.03
When I ride, my everyday worries fade away	123,681	−1.55	0.122	1,447	−0.04
I spend time practicing skills	124,968	−0.51	0.611	1,449	−0.01
I ride more casually	114,544	−2.47	0.014	1,444	−0.06
I enjoy bike maintenance	75,053	−11.18	0.000	1,445	−0.29
MTB is part of my identity	126,285	−0.31	0.760	1,447	−0.01
I suffer from mild mental health problems and use MTB as a coping strategy	108,266	−3.48	0.000	1,438	−0.09
I suffer from severe/enduring mental health problems and use MTB as a coping strategy	122,454	−0.72	0.473	1,440	−0.02
I would find it very depressing if I could no longer ride due to illness or injury	123,507	−1.76	0.078	1,447	−0.05
MTB is something I do to de-stress	124,196	−0.82	0.414	1,444	−0.02
MTB helps me to deal with negative thoughts and feelings	121,343	−1.62	0.105	1,447	−0.04
MTB makes me feel good about myself	119,500	−2.61	0.009	1,446	−0.07
I would stop MTBing if my friends stopped	126,407	−0.31	0.757	1,447	−0.01

*MTB, mountain biking*.

**Table 2:** Results from Mann–Whitney *U*-tests comparing *younger rider* and *older rider* responses.

	**Mann–Whitney *U***	***Z***	**Asymp. Sig. (2-tailed)**	***n***	***r***
I participated in MTB as a child	146,594	−13.90	0.000	1,467	−0.36
Level	235,138	−0.37	0.712	1,470	−0.01
Length of time participating	160,322	−10.78	0.000	1,470	−0.28
Frequency of participation (winter)	204,611	−4.54	0.000	1,466	−0.12
Frequency of participation (summer)	221,507	−1.26	0.209	1,441	−0.03
Perceived risk in personal participation	231,544	−0.80	0.423	1,469	−0.02
Preference (group or alone)	215,189	−3.25	0.001	1,471	−0.08
I participate in MTB for the adrenaline rush	205,395	−6.18	0.000	1,471	−0.16
MTB fulfills my need for adventure	220,453	−3.47	0.001	1,469	−0.09
I enjoy the risk inherent in MTB	214,913	−3.51	0.000	1,470	−0.09
I am not a risk taker	209,528	−3.86	0.000	1,465	−0.10
I like appearing adventurous to others	185,559	−7.61	0.000	1,466	−0.20
MTB encourages me to explore my local countryside	235,351	−0.63	0.528	1,469	−0.02
I love being outdoors	235,819	−1.39	0.165	1,470	−0.04
MTB allows me to feel more connected to nature and the world around me	227,362	−2.03	0.042	1,464	−0.05
Being outdoors helps me to de-stress	235,319	−1.37	0.171	1,469	−0.04
If the weather in bad, I don't go riding.	230,560	−0.97	0.332	1,467	−0.03
When I ride, my everyday worries fade away	228,449	−2.74	0.006	1,469	−0.07
I spend time practicing skills	193,142	−6.49	0.000	1,471	−0.17
I ride more casually	214,151	−3.24	0.001	1,466	−0.08
I enjoy bike maintenance	217,250	−3.06	0.002	1,467	−0.08
MTB is part of my identity	234,503	−0.57	0.569	1,469	−0.01
I suffer from mild mental health problems and use MTB as a coping strategy	215,064	−2.82	0.005	1,460	−0.07
I suffer from severe/enduring mental health problems and use MTB as a coping strategy	220,940	−2.39	0.017	1,460	−0.06
I would find it very depressing if I could no longer ride due to illness or injury	235,828	−0.51	0.609	1,462	−0.01
MTB is something I do to de-stress	233,671	−0.70	0.483	1,466	−0.02
MTB helps me to deal with negative thoughts and feelings	231,794	−1.10	0.271	1,469	−0.03
MTB makes me feel good about myself	227,958	−2.26	0.024	1,468	−0.06
I would stop MTBing if my friends stopped	233,761	−0.91	0.364	1,469	−0.02

*MTB, mountain biking*.

**Table 3:** Results from Mann–Whitney *U*-tests comparing *downhill rider* and *non-downhill rider* responses.

	**Mann–Whitney *U***	***Z***	**Asymp. Sig. (2-tailed)**	***n***	***r***
I participated in MTB as a child	197,666	−5.38	0.000	1,480	−0.14
Level	200,083	−5.05	0.000	1,483	−0.13
Age	186,948	−7.02	0.000	1,471	−0.18
Length of time participating	229,444	−0.54	0.588	1,483	−0.01
Frequency of participation (winter)	219,372	−1.85	0.064	1,479	−0.05
Frequency of participation (summer)	211,031	−2.57	0.010	1,454	−0.07
Perceived risk in personal participation	181,036	−7.88	0.000	1,482	−0.20
Preference (group or alone)	226,862	−1.00	0.317	1,484	−0.03
I participate in MTB for the adrenaline rush	201,172	−6.23	0.000	1,484	−0.16
MTB fulfills my need for adventure	221,964	−2.35	0.019	1,482	−0.06
I enjoy the risk inherent in MTB	185,534	−7.55	0.000	1,483	−0.20
I am not a risk taker	190,000	−6.05	0.000	1,478	−0.16
I like appearing adventurous to others	223,629	−1.31	0.190	1,479	−0.03
MTB encourages me to explore my local countryside	229,013	−1.19	0.235	1,482	−0.03
I love being outdoors	233,095	−0.16	0.875	1,483	0.00
MTB allows me to feel more connected to nature and the world around me	223,380	−2.03	0.042	1,477	−0.05
Being outdoors helps me to de-stress	232,183	−0.60	0.549	1,482	−0.02
If the weather in bad, I don't go riding	206,068	−4.06	0.000	1,484	−0.11
When I ride, my everyday worries fade away	230,131	−0.91	0.362	1,481	−0.02
I spend time practicing skills	183,169	−7.39	0.000	1,484	−0.19
I ride more casually	191,068	−5.98	0.000	1,479	−0.16
I enjoy bike maintenance	231,005	−0.30	0.764	1,480	−0.01
MTB is part of my identity	210,622	−4.86	0.000	1,482	−0.13
I suffer from mild mental health problems and use MTB as a coping strategy	211,400	−2.75	0.006	1,473	−0.07
I suffer from severe/enduring mental health problems and use MTB as a coping strategy	216,315	−2.42	0.016	1,475	−0.06
I would find it very depressing if I could no longer ride due to illness or injury	231,241	−0.62	0.538	1,482	−0.02
MTB is something I do to de-stress	227,977	−1.07	0.283	1,479	−0.03
MTB helps me to deal with negative thoughts and feelings	230,291	−0.55	0.581	1,482	−0.01
MTB makes me feel good about myself	226,749	−1.51	0.130	1,481	−0.04
I would stop MTBing if my friends stopped	228,949	−1.04	0.298	1,482	−0.03

*MTB, mountain biking*.

## Findings

### Participant characteristics

#### Objective participant characteristics

The characteristics of the 1,484 active international mountain bikers recruited are described in Table 4A. Men made up just over 80% of the sample. The most well represented age group was 36–45 years. Just over half of the participants considered themselves to be of intermediate standard. Approximately 40% indicated that they had been riding for over a decade. Nearly 60% indicated that they had not participated in mountain biking as a child.

**Table 4A:** Participant characteristics.

		**Total**		**DH**		**Non-DH**		**Male**		**Female**		**Younger riders (** ≤ **35)**		**Older riders (**>**35)**	
		***n*** = **1,484**		***n*** = **454**		***n*** = **1,030**		***n*** = **1,244**		***n*** = **205**		***n*** = **480**		***n*** = **991**	
		***n***	**%**	***n***	**%**	***n***	**%**	***n***	**%**	***n***	**%**	***n***	**%**	***n***	**%**
Gender	Female	205	13.8	50	11	155	15					66	13.8	139	14
	Male	1,244	83.8	392	86.3	852	82.7					405	84.4	834	84.2
	Prefer not to disclose	25	1.7	8	1.8	17	1.7					6	1.3	13	1.3
	Total	1,474	99.3	450	99.1	1,024	99.4					477	99.4	986	99.5
Age	16-25	145	9.8	68	15	77	7.5	132	10.6	10	4.9				
	26-35	335	22.8	137	30.2	198	19.2	273	21.9	56	27.3				
	36-45	538	36.6	165	36.3	373	36.2	456	36.7	77	37.6				
	46-55	369	25.1	71	15.6	298	28.9	302	24.3	54	26.3				
	56+	84	5.7	9	2	75	7.3	76	6.1	8	3.9				
	Total	1,471	99.1	450	99.1	1,021	99.1	1,239	99.6	205	100				
Level	Beginner	80	5.4	11	2.4	69	6.7	55	4.4	22	10.7	31	6.5	47	4.7
	Intermediate	844	56.9	233	51.3	611	59.3	687	55.2	139	67.8	268	55.8	570	57.5
	Advanced & Pro	559	37.7	210	46.3	349	33.9	502	40.4	43	21	181	37.7	373	37.6
	Total	1,483	99.9	455	100	1,029	99.9	1,244	100	204	99.5	480	100	990	99.9
Length of time as participant	Less than a year	68	4.6	17	3.7	51	5	49	3.9	17	8.3	33	6.9	34	3.4
	Between 1 and 5 years	503	33.9	154	33.9	349	33.9	403	32.4	88	42.9	235	49	263	26.5
	6–10 years	281	18.9	103	22.7	178	17.3	229	18.4	43	21	98	20.4	180	18.2
	Over 10 years	631	42.5	179	39.4	452	43.9	562	45.2	57	27.8	113	23.5	514	51.9
	Total	1,483	99.9	453	99.8	1,030	100	1,243	99.9	205	100	479	99.8	991	100
I participated in MTB as a child	Yes	603	40.6	231	50.9	372	36.1	552	44.4	41	20	318	66.3	280	28.3
	No	877	59.1	221	48.7	656	63.7	689	55.4	163	79.5	161	33.5	708	71.4
	Total	1,480	99.7	452	99.6	1,028	99.8	1,241	99.8	204	99.5	479	99.8	988	99.7

Participants were asked what type of mountain biking they had engaged in in the last 2 years, with the option of selecting more than one type (Table 5A). Those who ride cross-country and trails made up over 90% of the sample; over half have participated in enduro and all-mountain; approximately 30% in downhill; and pump track, 4X and freeride collectively made up <20% of the sample. Over a third of the participants indicated that they prefer to ride in groups compared with ~15% preferring to ride alone (Table 5A). Nearly half indicated that they have no preference. Approximately half of the participants indicated that the social aspect of mountain biking is important to them; however <4% of the participants indicated that they would stop engaging in mountain biking if their friends did not take part anymore (Table 5B).

Some disparities emerged when comparing the rider characteristics of males and females. Firstly, a Mann–Whitney test indicated that self-rated level was higher for males (*M* rank = 747) than for females (*M* rank = 586) (*U* = 98,695, *p* < 0.001, *r* = 0.15). To illustrate, ~20% of females indicated that they were advanced or professional riders compared with 40% of males. A Mann–Whitney test also indicated that males (*M* rank = 745) have been riding for longer than females (*M* rank = 599) (*U* = 101,654, *p* < 0.001, *r* = 0.13). Demonstrated descriptively, ~45% of males compared with 28% of females stated that they have been riding for more than a decade. Similarly, nearly 45% of males (*M* rank = 698) indicated they had participated in mountain biking as a child in comparison to only 20% of females (*M* rank = 874) *(U* = 95,718, *p* < 0.001 *r* = 0.14). The effect sizes of these differences are small, but of high statistical significance. Differences in the age range of males and females showed no statistical significance.

The participants were divided into two further independent samples: those who had taken part in downhill mountain biking in the past 2 years (*downhill riders*) and those who had not (*non-downhill riders*). A Mann–Whitney test revealed that there was a greater proportion of younger riders (*M* rank = 641) within the downhill rider sample (*U* = 186,947, *p* < 0.001, *r* = 0.18). A Mann–Whitney test also indicated that self-rated level of ability was higher for downhill riders (*M* rank = 816) than for non-downhill riders (*M* rank = 709), (*U* = 200,083, *p* < 0.001, *r* = 0.13), and similarly that a higher proportion of those who ride downhill (*M* rank = 664) also participated in mountain biking as a child (*U* = 197,666, *p* < 0.001, *r* = 0.14). Again, the effect sizes are small, but of high statistical significance. Downhill riders tented to ride more frequently, though the statistical significance is negligible.

Two additional independent samples were compared: those aged 35 years or younger (*younger riders*); and those aged 36 years or older (*older riders*). A Mann–Whitney test showed that older riders (*M* rank = 813) tended to have been riding for a longer period of time that younger riders (*M* rank = 575) (*U* = 160,321, *p* < 0.001), with a small-to-medium effect size (*r* = 0.28). Furthermore, approximately 66% of younger participants had ridden as a child, compared with just under 30% of participants over 35 years old. A Mann–Whitney test indicated that this had high statistical significance with a moderate effect size (*U* = 146,594, *p* < 0.001, *r* = 0.36).

#### Subjective participant characteristics

The subjective characteristics of the full sample and of the sub-sets are described in Table 4B. The majority of participants indicated that mountain biking forms part of their identity. The proportion in agreement was higher for downhill riders (*M* rank = 791) than for non-downhill riders (*M* rank = 720) (*U* = 210,622, *p* < 0.001, *r* = 0.13), but any differences between males and female or younger and older riders showed no statistical significance. Just over half of participants indicated that they wanted others to see them as adventurous, with a higher level of agreement for the younger riders (*M* rank = 840) in comparison to the older riders (*M* rank = 682) with a small effect size (*U* = 185,559, *p* < 0.001 *r* = 0.20).

**Table 4B:** Subjective participant characteristics.

		**Total**		**Downhill riders**		**Non-downhill riders**		**Male**		**Female**		**Younger riders (** ≤ **35)**		**Older riders (**>**35)**	
		***n*** = **1,484**		***n*** = **455**		***n*** = **1,030**		***n*** = **1,244**		***n*** = **205**		***n*** = **480**		***n*** = **991**	
		***n***	**%**	***n***	**%**	***n***	**%**	***n***	**%**	***n***	**%**	***n***	**%**	***n***	**%**
Mountain biking is part of my identity	Agree	1,275	85.9	419	92.3	856	83.1	1068	85.9	175	85.4	416	86.7	849	85.7
	Neutral	186	12.5	28	6.2	158	15.3	160	12.9	25	12.2	57	11.9	127	12.8
	Disagree	21	1.4	5	1.1	16	1.6	14	1.1	5	2.4	6	1.3	14	1.4
	Total	1,482	99.9	452	99.6	1,030	100	1,242	99.8	205	100	479	99.8	990	99.9
I want others to see me as an adventurous person	Agree	828	55.8	266	58.6	562	54.6	685	55.1	124	60.5	333	69.4	485	48.9
	Neutral	530	35.7	151	33.3	379	36.8	453	36.4	65	31.7	130	27.1	398	40.2
	Disagree	121	8.2	36	7.9	85	8.3	101	8.1	16	7.8	17	3.5	103	10.4
	Total	1,479	99.7	453	99.8	1,026	99.6	1,239	99.6	205	100	480	100	986	99.5
I do not consider myself to be a risk taker	Agree	310	20.9	61	13.4	249	24.2	249	20	52	25.4	79	16.5	230	23.2
	Neutral	418	28.2	111	24.4	307	29.8	355	28.5	54	26.3	126	26.3	290	29.3
	Disagree	750	50.5	280	61.7	470	45.6	634	51	99	48.3	275	57.3	465	46.9
	Total	1,478	99.6	452	99.6	1,026	99.6	1,238	99.5	205	100	480	100	985	99.4
I enjoy the element of risk or danger inherent in mountain biking	Agree	974	65.6	362	79.7	612	59.4	852	68.5	99	48.3	346	72.1	616	62.2
	Neutral	366	24.7	69	15.2	297	28.8	296	23.8	61	29.8	90	18.8	276	27.9
	Disagree	143	9.6	23	5.1	120	11.7	95	7.6	45	22	43	9	99	10
	Total	1,483	99.9	454	100	1,029	99.9	1,243	99.9	205	100	479	99.8	991	100

**Figure 1:**
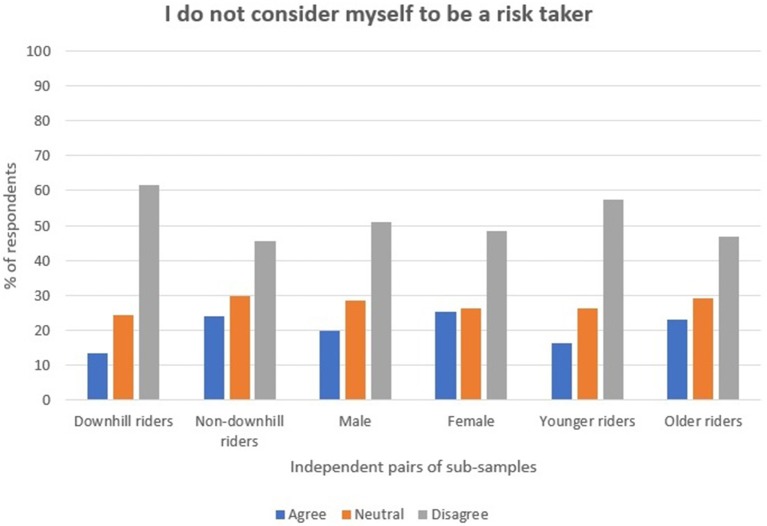
Participants' perception of themselves as risk takers.

**Figure 2:**
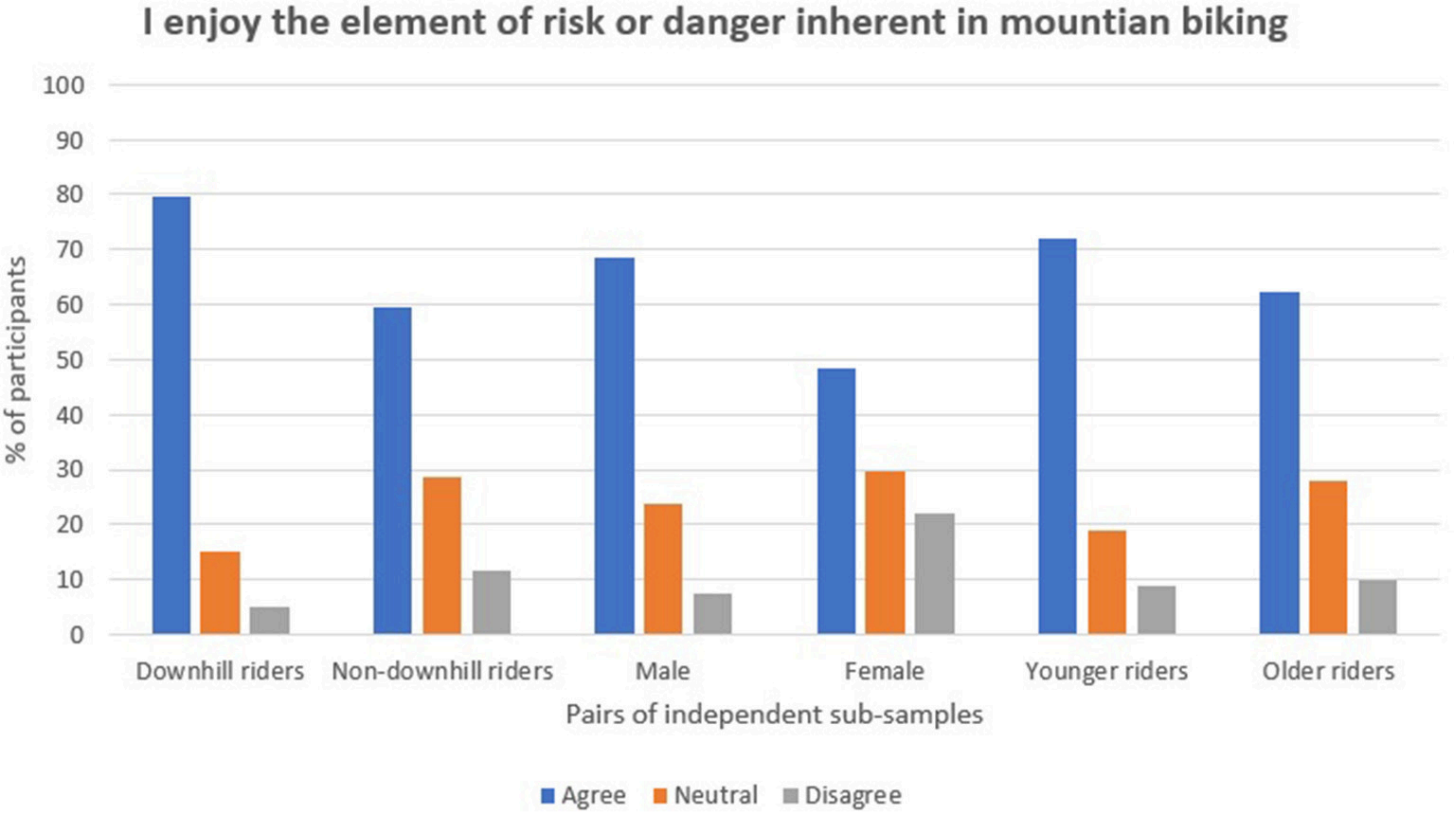
Participants' enjoyment of the risk or danger in mountain biking.

Regarding risk, just over 20% of the participants indicated that they did not consider themselves to be risk-takers, and approximately half indicated that they were risk-takers. A Mann–Whitney test showed that younger riders (*M* rank = 677) indicated that they were risk-takers at a higher rate than older riders (*M* rank = 760) (*U* = 209,527, *p* < 0.001, *r* = 0.10). Downhill riders (*M* rank = 647) were also more likely to consider themselves to be risk takers compared with the non-downhill rider sample (*M* rank = 780), (*U* = 190,000, *p* < 0.001, *r* = 0.16) (see Figure 1). Differences in the male and female responses showed little statistical significance. 65.6% in total agreed with the statement “I enjoy the element of risk or danger inherent in mountain biking.” Mann–Whitney tests indicated statistical significance in the differences in responses between all of the sub-samples: downhill riders (*M* rank = 848) showed a greater preference than non-downhill riders (*M* rank = 695) (*U* = 185,533, *p* < 0.001, *r* = 0.20); as did males (*M* rank = 748) compared with females (*M* rank = 580), (*U* = 97,843, *p* < 0.001, *r* = 0.17); and younger riders (*M* rank = 782) compared with older riders (*M* rank = 713), (*U* = 214,912, *p* < 0.001, *r* = 0.09) (see Figure 2).

### Engagement characteristics

Table 5A describes nominal data capturing various engagement characteristics, described in more detail in the following sections.

**Table 5:** Engagement characteristics.

		**Total**		**Downhill riders**		**Non-downhill riders**		**Male**		**Female**		**Younger riders (** ≤ **35)**		**Older riders (**>**35)**	
		***n*** = **1,484**		***n*** = **455**		***n*** = **1,030**		***n*** = **1,244**		***n*** = **205**		***n*** = **480**		***n*** = **991**	
		***n***	**%**	***n***	**%**	***n***	**%**	***n***	**%**	***n***	**%**	***n***	**%**	***n***	**%**
Riding frequency in Winter season	Twice a week or more	608	41	197	43.4	411	39.9	512	41.2	77	37.6	163	34	439	44.3
	Weekly to fortnightly	590	39.8	185	40.7	405	39.3	509	40.9	72	35.1	198	41.3	386	39
	Once a month or less	281	18.9	71	15.6	210	20.4	218	17.5	56	27.3	119	24.8	161	16.2
	Total	1,479	99.7	453	99.8	1,026	99.6	1,239	99.6	205	100	480	100	986	99.5
Riding frequency in Summer season	Twice a week or more	1,029	69.3	339	74.7	690	67	865	69.5	135	65.9	323	67.3	694	70
	Weekly to fortnightly	355	23.9	97	21.4	258	25	298	24	52	25.4	127	26.5	227	22.9
	Once a month or less	70	4.7	15	3.3	55	5.3	55	4.4	14	6.8	23	4.8	47	4.7
	Total	1,454	98	451	99.3	1,003	97.4	1,218	97.9	201	98	473	98.5	968	97.7
Type of MTB taken part in (in last 2 years)	Cross Country & T rail	1,379	92.9	396	87.2	983	95.4	1,148	92.3	198	96.6	431	89.8	936	94.5
	Enduro & All- Mountain	835	56.3	361	79.5	474	46	732	58.8	79	38.5	305	63.5	520	52.5
	Downhill	454	30.6	454	100	0	0	392	31.5	50	24.4	205	41.7	245	24.7
	Pump track, 4X, Freeride	277	18.7	181	39.9	96	9.3	240	19.3	31	15.1	127	26.5	147	14.8
	Total	1,484	100	454	100	1,030	100	1,244	100	205	100	480	100	991	100
Riding preference	Alone	224	15.1	42	9.3	182	17.7	194	15.6	25	12.2	64	13.3	158	15.9
	Group	540	36.4	188	41.4	352	34.2	440	35.4	82	40	206	42.9	329	33.2
	No preference	720	48.5	224	49.3	496	48.2	610	49	98	47.8	210	43.8	504	50.9
	Total	1,484	100	454	100	1,030	100	1,244	100	205	100	480	100	991	100

#### Riding frequency and style

Approximately 40% of the participants indicated that they ride twice a week or more in the winter season, and a similar number claimed to ride once or twice a fortnight (Table 5A). Participants reported riding more often in the summer season, with nearly 70% of participants claiming to ride twice a week or more. Summer season riding frequency was similar across all independent samples, however, Mann–Whitney tests indicated younger riders (*M* rank = 800) participated more frequently than older riders (*M* rank = 701) in the Winter (*U* = 185,533; *p* < 0.001, *r* = 0.12). Males and downhill riders also rode more frequently in the winter, though the statistical significance was slight.

#### Risk and skill-based engagement

**Figure 3:**
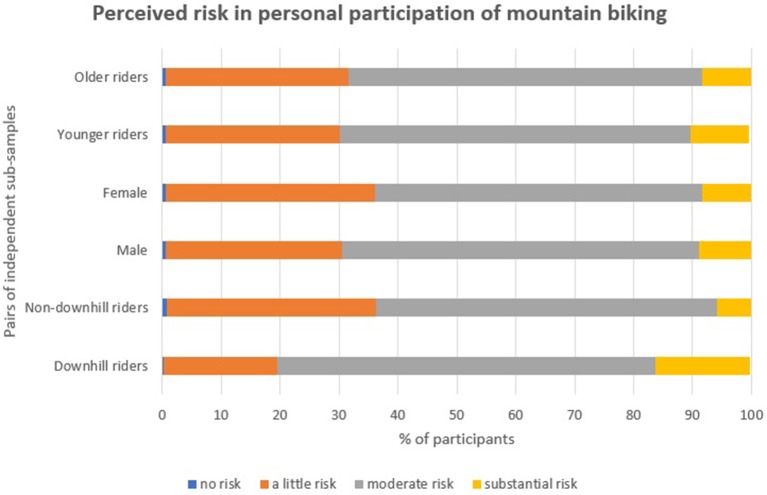
Participants' perceived risk in personal participation of mountain biking.

Regarding perceptions of risk; 0.5% of the participants indicated that there is “no risk” in their mountain biking participation; ~30% indicated “a little risk”; nearly 60% “moderate risk”; and 9% “substantial risk” (Table 5B). A Mann–Whitney test indicated that downhill riders (*M* rank = 856) considered there to be more risk in their participation than non-downhill riders (*M* rank = 691), (*U* = 181,036, *p* < 0.001, *r* = 0.20). To illustrate, ~16% of the downhill riders considered there to be “substantial risk” involved in comparison to just under 6% of the non-downhill riders (see Figure 3). Approximately 80% of the participants agreed that they engage in mountain biking because it gives them “an adrenaline rush.” Mann–Whitney tests indicated that a larger proportion of downhill riders (*M* rank = 814) agreed with this statement compared with non-downhill riders (*M* rank = 711), (*U* = 201,172, *p* < 0.001, *r* = 0.16); likewise, a larger proportion of younger riders (*M* rank = 804) compared with older riders (*M* rank = 703) agreed (*U* = 205,394, *p* < 0.001, *r* = 0.16).

**Table 5B:** Risk and sensation-seeking engagement characteristics.

		**Total**		**Downhill riders**		**Non-downhill riders**		**Male**		**Female**		**Younger riders (** ≤ **35)**		**Older riders (**>**35)**	
		***n*** = **1,484**		***n*** = **455**		***n*** = **1,030**		***n*** = **1,244**		***n*** = **205**		***n*** = **480**		***n*** = **991**	
		***n***	**%**	***n***	**%**	***n***	**%**	***n***	**%**	***n***	**%**	***n***	**%**	***n***	**%**
Perceived risk in personal participation	No risk	8	0.5	1	0.2	7	0.7	7	0.6	1	0.5	3	0.6	5	0.5
	A little risk	455	30.7	88	19.4	367	35.6	372	29.9	73	35.6	142	29.6	308	31.1
	Moderate risk	886	59.7	291	64.1	595	57.8	752	60.5	114	55.6	285	59.4	595	60
	Substantial risk	133	9	73	16.1	60	5.8	111	8.9	17	8.3	48	10	83	8.4
	Total	1,482	99.9	453	99.8	1,029	99.9	1,242	99.8	205	100	478	99.6	991	100
I participate in MTB because it gives me an adrenaline rush	Agree	1,195	80.5	409	90.1	786	76.3	1,016	81.7	154	75.1	431	89.8	755	76.2
	Neutral	238	16	40	8.8	198	19.2	192	15.4	38	18.5	41	8.5	194	19.6
	Disagree	51	3.4	5	1.1	46	4.5	36	2.9	13	6.3	8	1.7	42	4.2
	Total	1,484	100	454	100	1,030	100	1,244	100	205	100	480	100	991	100

Regarding skill-related engagement, ~20% of the total sample agreed with the statement “I do not concentrate on technical skills; I ride more casually.” A larger proportion of non-downhill riders (*M* rank = 780) agreed with this statement compared with downhill riders (*M* rank = 650), (*U* = 191,068, *p* < 0.001, *r* = 0.16) and similarly a larger proportion of older riders (*M* rank = 756) compared with younger riders (*M* rank = 687), (*U* = 214,151, *p* < 0.005 *r* = 0.08). Just over half of the participants stated that they regularly spend time learning and practicing technical skills. Downhill riders (*M* rank = 854) indicated that they spend more time practicing technical skills than non-downhill riders (*M* rank = 693), (*U* = 183,169, *p* < 0.001, *r* = 0.19); similarly, younger riders (*M* rank = 829) in comparison to older riders (*M* rank = 691), (*U* = 193,141, *p* < 0.001, *r* = 0.17). Just over 66% of the participants agreed with the statement “I enjoy the bike maintenance aspect of this activity” (Table 5C). A Mann–Whitney test showed that males (*M* rank = 765) indicated a stronger preference for bike maintenance activities in comparison to females (*M* rank = 470), (*U* = 75,052, *p* < .001), with a moderate effect size (*r* = 0.29). Similarly, but with a smaller effect size, younger riders (*M* rank = 774) indicated a stronger preference for bike maintenance in comparison to older riders (*M* rank = 714), (*U* = 217,250, *p* < 0.001, *r* = 0.08).

**Table 5C:** Skill-based engagement characteristics.

		**Total**		**Downhill riders**		**Non-downhill riders**		**Male**		**Female**		**Younger riders (** ≤ **35)**		**Older riders (**>**35)**	
		***n*** = **1,484**		***n*** = **455**		***n*** = **1,030**		***n*** = **1,244**		***n*** = **205**		***n*** = **480**		***n*** = **991**	
		***n***	**%**	***n***	**%**	***n***	**%**	***n***	**%**	***n***	**%**	***n***	**%**	***n***	**%**
I do not concentrate on technical skills; I ride more casually	Agree	305	20.6	55	12.1	250	24.3	243	19.5	54	26.3	79	16.5	225	22.7
	Neutral	410	27.6	117	25.8	293	28.4	344	27.7	60	29.3	128	26.7	280	28.3
	Disagree	764	51.5	281	61.9	483	46.9	652	52.4	91	44.4	273	56.9	481	48.5
	Total	1,484	100	453	99.8	1,026	99.6	1,239	99.6	205	100	480	100	986	99.5
I regularly spend time learning/practicing technical skills	Agree	796	53.6	307	67.6	489	47.5	664	53.4	111	54.1	317	66	469	47.3
	Neutral	462	31.1	109	24	353	34.3	402	32.3	53	25.9	110	22.9	350	35.3
	Disagree	226	15.2	38	8.4	188	18.3	178	14.3	41	20	53	11	172	17.4
	Total	1,484	100	454	100	1,030	100	1,244	100	205	100	480	100	991	100
I enjoy the Bike maintenance of this activity	Agree	986	66.4	307	67.6	679	65.9	889	71.5	71	34.6	346	72.1	631	63.7
	Neutral	282	19	78	17.2	204	19.8	222	17.8	56	27.3	74	15.4	205	20.7
	Disagree	121	14.3	69	15.2	143	13.9	130	10.5	77	37.6	59	12.3	152	15.3
	Total	1,484	100	454	100	1,026	99.6	1,241	99.8	204	99.5	479	99.8	988	99.7

### Perceived well-being outcomes

Participants completed a range of questions related to the mountain biking environment (Table 6A). Almost 100% of the participants agreed that they “love being outdoors,” and over 98% of the participants indicated that being outdoors helps them to de-stress. Nearly 90% of the participants agreed that mountain biking makes them feel more connected to nature and the world around them. Over 90% of the participants agreed that participating in mountain biking has made them more likely to explore their local countryside. Mann–Whitney tests revealed that these figures remained stable irrespective of the confounding factors of age, gender or type of rider. Approximately 12% of the total sample agreed that they do not ride if the weather is bad. A Mann–Whitney test showed that a higher proportion of non-downhill riders (*M* rank = 766) choose not to ride in bad weather in comparison to downhill riders (*M* rank = 682), (*U* = 206,068, *p* < 0.001, *r* = 0.11).

**Table 6A:** The mountain biking environment.

		**Total**		**Downhill riders**		**Non-downhill riders**		**Male**		**Female**		**Younger riders (** ≤ **35)**		**Older riders (**>**35)**	
		***n*** = **1,484**		***n*** = **455**		***n*** = **1,030**		***n*** = **1,244**		***n*** = **205**		***n*** = **480**		***n*** = **991**	
		***n***	**%**	***n***	**%**	***n***	**%**	***n***	**%**	***n***	**%**	***n***	**%**	***n***	**%**
Being outdoors helps me to de-stress	Agree	1,463	98.6	446	98.2	1,017	98.7	1,225	98.5	204	99.5	471	98.1	979	98.8
	Neutral	17	1.1	6	1.3	11	1.1	15	1.2	1	0.5	9	1.9	8	0.8
	Disagree	2	0.1	1	0.2	1	0.1	2	0.2	0	0	0	0	2	0.2
	Total	1,482	99.9	453	99.8	1,029	99.9	1,242	99.8	205	100	480	100	989	99.8
Mountain biking makes me feel more connected to nature & the world around me	Agree	1,317	88.7	392	86.3	925	89.8	1,095	88	189	92.2	415	86.5	891	89.9
	Neutral	148	10	54	11.9	94	9.1	135	10.9	11	5.4	59	12.3	87	8.8
	Disagree	12	0.8	6	1.3	6	0.6	10	0.8	2	1	4	0.8	8	0.8
	Total	1,477	99.5	452	99.6	1,025	99.5	1,240	99.7	202	98.5	478	99.6	986	99.5
Mountain biking has made me more likely to explore my local countryside	Agree	1,390	93.7	419	92.3	971	94.3	1,169	94	189	92.2	453	94.4	925	93.3
	Neutral	65	4.4	21	4.6	44	4.3	51	4.1	12	5.9	19	4	45	4.5
	Disagree	27	1.8	12	2.6	15	1.5	22	1.8	4	2	8	1.7	19	1.9
	Total	1,482	99.9	452	99.6	1,030	100	1,242	99.8	205	100	480	100	989	99.8
If the weather is bad, I won't go riding	Agree	180	12.1	36	7.9	144	14	156	12.5	21	10.2	50	10.4	128	12.9
	Neutral	365	24.6	98	21.6	267	25.9	310	24.9	49	23.9	121	25.2	243	24.5
	Disagree	935	63	318	70	617	59.9	775	62.3	134	65.4	309	64.4	616	62.2
	Total	1,484	100	452	99.6	1,028	99.8	1,241	99.8	204	99.5	480	100	987	99.6
I love being outdoors	Agree	1,469	99	449	98.9	1,020	99	1,230	98.9	205	100	473	98.5	983	99.2
	Neutral	13	0.9	3	0.7	10	1	12	1	0	0	7	1.5	6	0.6
	Disagree	1	0.1	1	0.2	0	0	1	0.1	0	0	0	0	1	0.1
	Total	1,483	99.9	453	99.8	1,030	100	1,243	99.9	205	100	480	100	990	99.9

Participants also completed a range of questions regarding perceived well-being outcomes in relation to their engagement in mountain biking (Table 6B). Nearly 90% of participants agreed that mountain biking makes them feel good about who they are. Females, younger riders and downhill riders tended to agree at a higher rate though statistical significance was negligible. Over 80% agreed that participating in mountain biking helps them to deal with negative thoughts or feelings, and over 90% indicated that it is something they do to de-stress; figures that remained stable across the independent sub-samples. Approximately 93% of participants agreed with the statement “when I ride my everyday worries fade away,” and nearly 95% of the total participants stated that they would find it depressing if they could no longer ride due to illness or injury; again, with the figures remaining steady across sub-samples.

**Table 6B:** Mountain Biking and well-being.

		**Total**		**Downhill riders**		**Non-downhill riders**		**Male**		**Female**		**Younger riders (** ≤ **35)**		**Older riders (**>**35)**	
		***n*** = **1,484**		***n*** = **455**		***n*** = **1,030**		***n*** = **1,244**		***n*** = **205**		***n*** = **480**		***n*** = **991**	
		***n***	**%**	***n***	**%**	***n***	**%**	***n***	**%**	***n***	**%**	***n***	**%**	***n***	**%**
Participating in mountain biking makes me feel good about who I am	Agree	1,326	89.4	414	91.2	912	88.5	1,099	88.3	194	94.6	442	92.1	872	88
	Neutral	146	9.8	35	7.7	111	10.8	134	10.8	10	4.9	37	7.7	108	10.9
	Disagree	9	0.6	4	0.9	5	0.5	8	0.6	1	0.5	1	0.2	8	0.8
	Total	1,481	99.8	453	99.8	1,028	99.8	1,241	99.8	205	100	480	100	988	99.7
Mountain biking helps me to deal with negative thoughts or feelings	Agree	1,219	82.1	377	83	842	81.7	1,013	81.4	177	86.3	402	83.8	806	81.3
	Neutral	234	15.8	64	14.1	170	16.5	205	16.5	24	11.7	71	14.8	161	16.2
	Disagree	29	2	12	2.6	17	1.7	24	1.9	4	2	7	1.5	22	2.2
	Total	1,482	99.9	453	99.8	1,029	99.9	1,242	99.8	205	100	480	100	989	99.8
Mountain biking is something I do to de -stress	Agree	1,339	90.2	415	91.4	924	89.7	1,120	90	188	91.7	437	91	889	89.7
	Neutral	118	8	29	6.4	89	8.6	102	8.2	13	6.3	35	7.3	83	8.4
	Disagree	22	1.5	8	1.8	14	1.4	18	1.4	3	1.5	7	1.5	15	1.5
	Total	1,479	99.7	452	99.6	1,027	99.7	1,240	99.7	204	99.5	479	99.8	987	99.6
When I ride my everyday worries fade away	Agree	1,387	93.5	428	94.3	959	93.1	1,158	93.1	197	96.1	461	96	913	92.1
	Neutral	88	5.9	22	4.8	66	6.4	78	6.3	7	3.4	19	4	69	7
	Disagree	7	0.5	3	0.7	4	0.4	6	0.5	1	0.5	0	0	7	0.7
	Total	1,482	99.9	453	99.8	1,029	99.9	1,242	99.8	205	100	480	100	989	99.8
I would find it depressing if could no longer ride due to illness or injury	Agree	1,402	94.5	431	94.9	971	94.3	1,181	94.9	189	92.2	456	95	933	94.1
	Neutral	50	3.4	14	3.1	36	3.5	41	3.3	7	3.4	14	2.9	36	3.6
	Disagree	30	2	8	1.8	22	2.1	20	1.6	9	4.4	10	2.1	20	2
	Total	1,482	99.9	453	99.8	1,029	99.9	1,242	99.8	205	100	480	100	989	99.8

Participants responded to questions directly related to pre-existing and self-defined mental health problems (Table 6C). 33.8% of the participants agreed with the statement “I have mild mental health problems (such as stress, depression, or anxiety) and use mountain biking as a coping strategy.” A Mann-Whitney test indicated that the difference between the female (*M* rank = 806) and male (*M* rank = 705) responses was statistically significant (*U* = 108,266, *p* < 0.001), though with small effect size (*r* = 0.09). Younger riders and downhill riders also agreed with this statement at a slightly higher rate, though with minor statistical significance and small effect size. 7.3% of the participants agreed with the statement “I suffer from severe or enduring mental health problems and use mountain biking as a coping strategy.” Again, younger riders and downhill riders agreed with this statement at a higher rate, though with low statistical significance and effect size. Male and female responses displayed little variance.

**Table 6C:** Active use of mountain biking as a coping strategy.

		**Total**		**Downhill riders**		**Non-downhill riders**		**Male**		**Female**		**Younger riders (** ≤ **35)**		**Older riders (**>**35)**	
		***n*** = **1,484**		***n*** = **455**		***n*** = **1,030**		***n*** = **1,244**		***n*** = **205**		***n*** = **480**		***n*** = **991**	
		***n***	**%**	***n***	**%**	***n***	**%**	***n***	**%**	***n***	**%**	***n***	**%**	***n***	**%**
I suffer from mild mental health problems (e.g., stress, anxiety, or depression) and use MTB as a coping strategy	Agree	502	33.8	173	38.1	329	31.9	393	31.6	94	45.9	185	38.5	315	31.8
	Neutral	269	18.1	87	19.2	182	17.7	235	18.9	30	14.6	90	18.8	177	17.9
	Disagree	702	47.3	191	42.1	511	49.6	606	48.7	80	39	203	42.3	490	49.4
	Total	1,473	99.3	451	99.3	1,022	99.2	1,234	99.2	204	99.5	478	99.6	982	99.1
I suffer from severe/enduring mental health problems and use MTB as a coping strategy	Agree	109	7.3	44	9.7	65	6.3	89	7.2	18	8.8	41	8.5	67	6.8
	Neutral	305	20.6	100	22	205	19.9	256	20.6	43	21	113	23.5	191	19.3
	Disagree	1,061	71.5	306	67.4	755	73.3	892	71.7	142	69.3	324	67.5	726	73.3
	Total	1,475	99.4	450	99.1	1,025	99.5	1,237	99.4	203	99	478	99.6	984	99.3

### Summary of significant differences within the sub-samples

#### Male and female riders

Our results show that males tended to have been riding for longer than females and rated themselves at a higher ability level. Males were also more likely to have ridden as children. Males indicated that they enjoy the risk involved more than females, as well as the bike maintenance aspect of the activity. Females indicated that mountain biking helped them to deal with mild mental health difficulties at a higher rate than men. All of these differences showed only small effect sizes, with the exception of the male preference for bike maintenance, which displayed a moderate effect size. Differences between the genders displayed the least variability amongst the three sub-samples.

#### Younger and older riders

Younger riders were more likely to have ridden as children, participate more in the winter, and practice skills more often than older riders. Younger riders were also more likely than older riders to consider themselves to be risk-takers, want others to see them as adventurous, be motivated by the “adrenaline rush,” and enjoy the risk and danger inherent in mountain biking. Furthermore, younger riders showed a preference for bike maintenance activities. Older riders indicated that they had been riding for longer than younger riders, and were more likely to ride more casually. The differences displayed small to medium effect sizes.

#### Downhill riders and non-downhill riders

Downhill riders tended to be younger, were more likely to have ridden as children, and indicated that they were at a more proficient ability level than non-downhill riders. Downhill riders indicated that they spend more time practicing skills and are more likely to ride even when the weather is bad in comparison to non-downhill riders; who were conversely more likely to state they ride more casually. Furthermore, downhill riders expressed that there is more risk involved in their participation than non-downhill riders, and they are more likely to consider themselves to be risk-takers as well as enjoying the risk and danger involved. Downhill riders were also more motivated to engage for an “adrenaline rush” than non-downhill riders, and more likely to state that mountain biking forms part of their identity. The differences displayed small to medium effect sizes.

## Discussion

This study aimed to describe the characteristics of mountain biking participants, their engagement methods, and perceived benefits of mountain biking on mental health and well-being. Our findings have succeeded in describing the overall sample as well as highlighting differences and similarities between certain paired groups of mountain bikers. Initially, this discussion elaborates on the characteristics of the participants and their engagement methods. Subsequently, we draw upon these findings to support our discussion of the engagement methods and rider attitudes related to risk and skill; the disproportion of female to male riders; and perceived well-being benefits.

### Participant characteristics and engagement

Our results regarding gender and age are congruent with other similar studies (e.g., Skår et al., 2008; Cycling UK, 2017; Hill et al., 2017); and confirm that mountain biking is a male-dominated sport, largely performed by those aged 36–55. A significantly higher percentage of the younger riders engaged in mountain biking as a child compared with the older riders, lending weight to the claim that mountain biking has boomed in popularity over the last 30 years (Moularde and Weaver, 2016; Poulson, 2016). Trail riding and cross-country were the most popular methods of engaging in mountain biking, with downhill one of the least common, again mirroring recent findings (Cycling UK, 2017). Participants generally reported having been involved in mountain biking for many years, in particular the older riders; indicating that they participate regularly throughout the year. Again, this is congruent with other findings (Taylor, 2010; Cycling UK, 2017). Many studies have highlighted the central importance of the social aspect of mountain biking and similar sports (e.g., Willig, 2008; International Mountain Biking Association, 2010; Moularde and Weaver, 2016; Cycling UK, 2017; Frühauf et al., 2017). Our findings show that although the social aspect is important; there is some apathy regarding riding alone or with others, and <4% claimed they would stop participating if their friends stopped. Further study is needed; however, we suggest that although camaraderie may be a motivator to a certain extent, it is not a vital aspect of the activity.

### Attitudes to risk

The mountain bikers surveyed displayed variability in their perception and experience of risk, supporting recent studies (e.g., Jones et al., 2015; Lynch and Dibben, 2016; Brymer and Schweitzer, 2017; Cycling UK, 2017). Younger riders were more likely to enjoy the level of risk and danger, view themselves as risk-takers, as well as being more motivated by the adrenaline rush, compared with older riders. Younger riders were also more likely to spend time practicing technical skills. The study highlighted similar findings amongst downhill riders. Downhill riding is one of the most high-risk methods of engaging in mountain biking (Becker et al., 2013; Hagen, 2013; Becker and Moroder, 2017). Predictably, those who have recently participated in downhill mountain biking were more likely to indicate a higher level of risk in their participation than those who had not. Downhill riders, in a similar way to younger riders, were also more likely to enjoy the risk involved, consider themselves to be risk-takers, and be motivated by an “adrenaline rush.” To be able to engage relatively safely in an activity that poses significant risk such as downhill riding, one must mitigate against the risk. One way of doing this is to ensure that relevant skills are developed sufficiently through practice and commitment to the activity. Downhill riders did tend to indicate that they were of a higher level of ability and were more likely to spend time practicing technical skills than those who do not participate in downhill riding. They were also slightly more likely to ride more often and in all weather. These findings are compatible with the theory that participants of extreme or adventure sports are capable of matching their skill level to their method of engagement (Llewellyn and Sanchez, 2008; Taylor, 2010; Lynch and Dibben, 2016; Frühauf et al., 2017); and they typically ensure that risks are within personal capabilities (Willig, 2008; Jones et al., 2015). Crucially, these younger riders and downhill riders suggest they experience something pleasurable from the experience of high-risk engagement. It is possible that an awareness of danger contributes to the value of the extreme sport experience for some individuals; that the tangible presence of risk adds to a sense of pushing personal limitations, contributing to self-esteem and confidence (Willig, 2008; Taylor, 2010). Similarly, Jones et al. (2015) argues that facing a challenge on the edge of one's abilities can provide the participant with a sense of gratification and increase self-efficacy. As such, experiencing enjoyment alongside fear may not be as strange or negative a phenomenon as it outwardly appears (Dodson, 1996; Lyng, 2005; Willig, 2008). A detailed, precise study concentrating on the benefits of perceived risk-taking in mountain biking could further enhance this line of argument.

Seemingly inconsistent with the assumption that males take more risks than females (Byrnes et al., 1999; Weber et al., 2002); males and females in this study did not differ in their perception of themselves as risk-takers or of the risk involved in their participation. Neither did they differ in how much they are motivated by an adrenaline rush. A more nuanced finding, however, is that males, similar to younger riders and downhill riders, were more likely to indicate that they enjoy the level of perceived risk. This suggests that although subjective risk-taking behavior is analogous across both genders; male mountain bikers appear to be slightly more comfortable with the perceived risk or danger. This could support Weber et al.'s (2002) claim that men generally expect more benefits to arise following risk taking, including within recreational domains. We can again incorporate the concept of enactive task-mastery here; that is, positive experiences that occur through successfully testing one's skills in challenging situations. Our study found that male mountain bikers tended to have been riding for longer, consider themselves more advanced, and were more likely to have ridden as children. It is arguable therefore that these factors could be affording men more opportunities for experiences of enactive task-mastery; that is, providing them with more lived experience of the benefits which are realized by pushing ones limits and taking measured risks within the sport. Again, a more nuanced study would further enrich this study's preliminary findings.

### Gender disparity

The opinions and experiences of males and females did not vary substantially. There were some differences however: males considered themselves to be at a higher level of riding ability, had been riding for longer, and were more likely to have ridden as children in comparison to females. Males also indicated that they enjoyed the risk and danger involved more than females, as discussed above. The most substantial variance however was regarding attitudes toward bike maintenance. There is a dearth of peer-reviewed literature regarding females and their attitudes to bike maintenance or mechanics, however, the popular press often states that female cyclists can find the mechanical world of bikes intimidating, and often lack the confidence to engage in this aspect of cycling (Milley, 2016). As such, females may not be experiencing the full freedom that cycling offers. Mountain biking is continually developing as a sport, largely due to technical innovation (Huybers-Withers and Livingston, 2010), and so if females dislike or are uncomfortable with the technical side, integral aspects of mountain biking may remain foreign to them. Furthermore, important (female) insights may be overlooked by the industry, hindering the development of this ever-evolving sport (Huybers-Withers and Livingston, 2010). Results from our study suggest that females are less likely than males to participate in mountain biking as a child; it is possible, therefore, that females are also less likely to have engaged in any form of bike maintenance as a child. Female bike mechanics have argued that because girls are not taught how to fix their bikes when they are younger, they lack the confidence to learn these skills in later life^1^ Female-only skill-based groups, workshops and scholarships are opening up across the globe in order to tackle this, where women are supported to learn new skills in empathetic learning environments.

These findings are only preliminary; however, it is arguable that the current gender disparity in this activity starts at a young age, when girls are less likely to participate in mountain biking than boys. This could also provide part of the explanation as to why females rate themselves at a lower ability level and are more likely to dislike bike maintenance—they may lack confidence in learning a new and “foreign” skill; one that males are often more familiar with. It is possible that encouraging more girls to engage in all aspects of mountain biking when they are younger may not only tackle the gender disparity, but also increase the proportion of female riders who feel confident and competent in their riding ability and enjoy all aspect of the sport. Further precise research is required to enable additional investigation.

### Nature

Regular exercise is known to be integral to health and well-being, and outdoor environments are consistently found to be more therapeutic than indoor environments (Hug et al., 2009; Ryan et al., 2010; Scheinfeld et al., 2011; Mitchell, 2013; Martyn and Brymer, 2016; Yeh et al., 2016). Evidence suggests that maintenance of a positive mood requires adherence to long term physical activity (Mead et al., 2009). The participants in our study were motivated to participate on a regular basis over a prolonged period with little variance across the subsamples; thus arguably increasing the likelihood of sustaining emotional balance. The participants, almost unanimously, loved being outdoors and recognized its therapeutic potential to deal with stress. This supports findings that the outdoor environment acts as a significant motivator for regular, sustained participation in sports such as mountain biking (International Mountain Biking Association, 2010; Taylor, 2010; Davidson and Stebbins, 2011; Lynch and Dibben, 2016; Moularde and Weaver, 2016). Our findings cannot definitively tell us whether participants would exercise as regularly (if at all) if they were not able to participate in mountain biking. Our data does show, however, that mountain biking encourages all riders to get outside and explore their local countryside more; and this did not show any variability across any subsamples. It is arguable that these participants would not be accessing the outdoors as much were it not for their participation in mountain biking.

The feeling of connection with nature is thought to be of great benefit to human well-being (Martyn and Brymer, 2016). Our results confirm that mountain biking provides opportunities for participants to feel this connection, supporting Davidson and Stebbins (2011) assertion that it is common for adventure sport participants to talk about feeling that they are “part of nature” (p. 183). Downhill riders in Hagen's (2013) qualitative study did not mention any well-being benefits afforded by riding in wilderness environments; though they did talk about connections with specific trails or obstacles. In contrast, our study showed no meaningful variance in the responses from those who rode downhill compared to those who did not with regards to feeling an improved connection to nature through riding. Participants in Hagen's (2013) study were all elite riders, which may have influenced her results; and furthermore, many of the downhill riders in our study also rode cross-country and on trails.

Only a very small minority of participants indicated that they did not ride in bad weather. Exercising in changeable outdoor environments is thought to provide anxiolytic effects as participants become “more comfortable with uncomfortable somatic and sensory experiences,” similar to the physical symptoms of anxiety (Lawton et al., 2017, p. 8). This is especially true of exercise in adverse or uncomfortable conditions (Anderson and Shivakumar, 2013). Countless mountain bikers may therefore be experiencing additional mental health benefits by riding in less favorable conditions. The majority of mountain bikers in our survey acknowledged that mountain biking maintained a sense of emotional balance: helping them to de-stress and deal with negative thoughts and everyday worries; and encouraging them to feel good about themselves. However, whether or not these concepts translate into an improved ability to deal with symptoms of anxiety in everyday life was not captured. Furthermore, the precise mechanisms by which mountain biking improves perceived well-being were not sufficiently measured, therefore we cannot say to what degree our findings are due to the environment within which mountain biking occurs or to something else integral to the activity.

### Mental health and well-being

Participants reported many positive mental health benefits directly linked to their participation in mountain biking, including an improved mood, a decrease in stress and worry, and increased self-esteem. It is vital to note that the well-being outcomes measured in this study showed minimal variability across all subsamples, despite the variability in engagement methods and characteristics of riders within the subsamples. This is supported, in part, by findings from Pretty et al. (2007), whereby the mood and self-esteem outcomes of participants of 10 different outdoor leisure activities did not differ according to type of activity, or its duration or intensity. Mountain biking is versatile, with a myriad of ways in which to participate. It could be surmised that mountain bikers are able to locate themselves within this activity and participate in a way which matches their motivations and character traits, thus allowing them to benefit equally.

Participation in high-risk sports can act as a means by which individuals manage their emotional life and alleviate negativity (Taylor, 2010; Brymer and Schweitzer, 2013). Indeed, our study illustrates that many mountain bikers believe that they would be depressed if they could no longer ride. This suggests that mountain biking plays a significant role in the maintenance of positive mental health and well-being for its participants, supporting findings by Cycling UK (2017). Willig (2008) paints a similar picture, whereby individuals experience their participation in high-risk sports as a fundamental need that helps them to function in everyday life—some participants went as far as to state that they would be using alcohol or drugs in order to meet their emotional needs were it not for extreme sports. Depression, the leading cause of ill health worldwide, increases the risk of substance misuse substantially (World Health Organization, 2018); clearly presenting far greater risks of injury and death than even the most extreme of sports.

One in three participants in our study stated they experience common mental health problems, with women being disproportionately affected, and use mountain biking as a coping strategy. This conforms to recent figures published by the World Health Organization (World Health Organization, 2018). Men are known to be less likely to both indicate that they have mental health problems and seek professional psychological help, unless they are very severely distressed (Mackenzie et al., 2006; World Health Organization, 2018). Unsustainable and damaging coping strategies, often leading to alcohol dependence and drug misuse, are more common for men (World Health Organization, 2018). Male mental ill-health, especially at the less severe end of the spectrum, may materialize in ways that do not fit as easily within conventional approaches to treatment. It is a crucial point that such a large proportion of our survey participants proactively use mountain biking as a coping strategy. Adding strength to this is that a slightly larger proportion of males compared with females claimed they would be depressed if they could no longer engage in mountain biking; a recognition that mountain biking could be acting as a substantial protective factor in their lives. Outdoor adventurous activities are likely to have lasting benefit to mental health (Brymer et al., 2010; Ryan et al., 2010; Mitchell, 2013; Martyn and Brymer, 2016; Yeh et al., 2016; Lawton et al., 2017). In the context of a society within which most people experiencing psychological disorders and/or emotional distress remain both undiagnosed and untreated (Mental Health Taskforce, 2016; World Health Organization, 2018), it is imperative that individuals, in particular those less likely to seek professional help, find ways to mediate and maintain their own mental health. The use of proactive and unconventional interventions, such as mountain biking, need careful consideration and further research.

## Limitations

A proportion of the participants stated they like others to see them as adventurous people, resonating with Willig's (2008) insights into identity formation in extreme sports. This serves as a warning for social desirability bias; however, due to the survey being online and anonymous the bias is likely to be limited. By exclusively recruiting online, self-selection bias is increased, and could be skewed toward those who participate regularly or have a disproportionate level of enthusiasm for the sport. There are also limitations associated with cross-sectional retrospective data, which relies on participant recall.

A non-standardized survey tool was used for data collection. Although this allowed an expansive range of data to be captured related specifically to mountain biking; it also restricted the possible rigor of well-being outcome measurements. Future research could utilize qualitative methodologies in order to capture and explore some of the subtleties highlighted within this study; or standardized measures such as the Benefits of Hiking Scale, adapted for use with mountain biking by Hill et al. (2017), or the self-esteem and psychological health measures used in Pretty et al's (2007) study. The development of Likert Scales as opposed to using single Likert items could further enhance the potential for stronger conclusions in future studies.

## Conclusions and future directions

Firstly, this study's findings, alongside other evidence, suggests that risk-taking behavior in this field does not necessarily have negative connotations. This study suggests that those who are more motivated by risk (in particular, younger, downhill riders) are also more likely to enjoy this aspect of the sport; and as such are also more inclined to mitigate against these risks by spending more time practicing the required skills to deal with those situations, and arguably being more committed to the sport (riding more often and in all weathers). Further qualitative research would better develop the subtleties discussed with regard to risk-taking attitudes and behaviors of mountain bikers implied within this study; however, our findings imply that the experience of risk can be positive and enjoyable, and may motivate participants to commit time and effort to master skills.

Secondly, our findings confirm that females are under-represented in mountain biking, though the majority of their experiences, motivations, preferences and perceived well-being outcomes vary very little when compared with males. In conjunction with existing literature, we suggest that in order to begin to address this disparity, women ought to be encouraged to be involved in all aspects of the sport from a younger age. This is important because a high percentage of the females in this study report that this activity helps them to regulate their mental health; and as such, it could be helping others. Rigorous and specific research is required in order to expand upon this recommendation.

Finally, these findings confirm that mountain bikers are not a homogeneous group; rather the population is composed of people who not only participate in this activity in many different ways, but also have different characteristics, motivations, preferences, habits and styles. The study did suggest that younger riders were slightly more likely to be drawn toward the “riskier” ways of participating in mountain biking, and were also more committed to the skills-based aspect of the sport; and that older riders who did not participate in downhill riding rode more casually. It is a tribute to the versatility and malleability of mountain biking that so many diverse participants are able to engage in ways which appear to suit them. Importantly, mountain bikers reported copious benefits to mental health and well-being related to their engagement, with minimal variability across all participants and irrespective of the confounding factors related to rider characteristics or engagement methods. A large proportion of the participants currently use mountain biking as a coping mechanism for their pre-existing mental health problems; and the majority use it as a way to deal with stress. As such, our findings provide insights that could inform future research, which could then encourage the development of outdoor adventure activities such as mountain biking as complementary mental health interventions; particularly for those who are less likely to access conventional therapies.

## Author's note

LR was a member of the NIHR/HEE Integrated Clinical Academic Programme Internship Scheme.

## Author contributions

LR: design, data collection, analysis, full write up; GJ: assistance with introduction, style and statistical analysis; RB: assistance with design, data collection, literature search, introduction, discussion, style.

### Conflict of interest statement

The authors declare that the research was conducted in the absence of any commercial or financial relationships that could be construed as a potential conflict of interest.

## Footnotes

^1^https://www.cyclinguk.org/blog/victoria-hazael/day-women-bike.
