# Supplementary material for: Why Do You Ride?: A Characterization of Mountain Bikers, Their Engagement Methods, and Perceived Links to Mental Health and Well-Being
Source: Front Psychol. 2018 Sep 19;9:1642. doi: 10.3389/fpsyg.2018.01642 (PMC6156442; doi:10.3389/fpsyg.2018.01642)

**Appendix 1: Copy of full survey tool.**

The platform SnapSurveys was used for data collection (http://www.snapsurveys.com/).

Two surveys were live during the data collection phase due to a spelling mistake in the first.

A summary of Survey 1 is reproduced below, which displays all questions and response rates for this version of the survey.


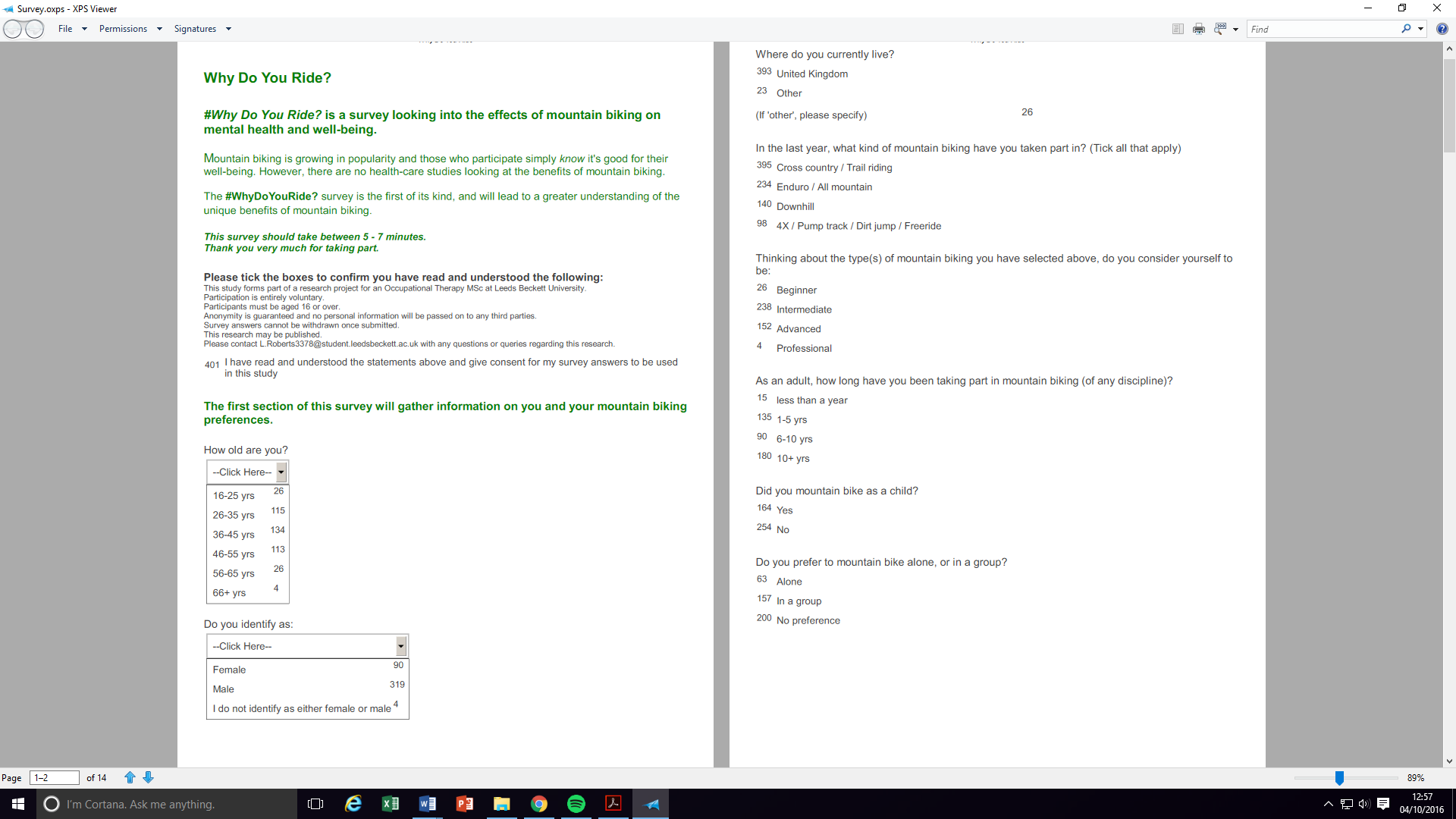


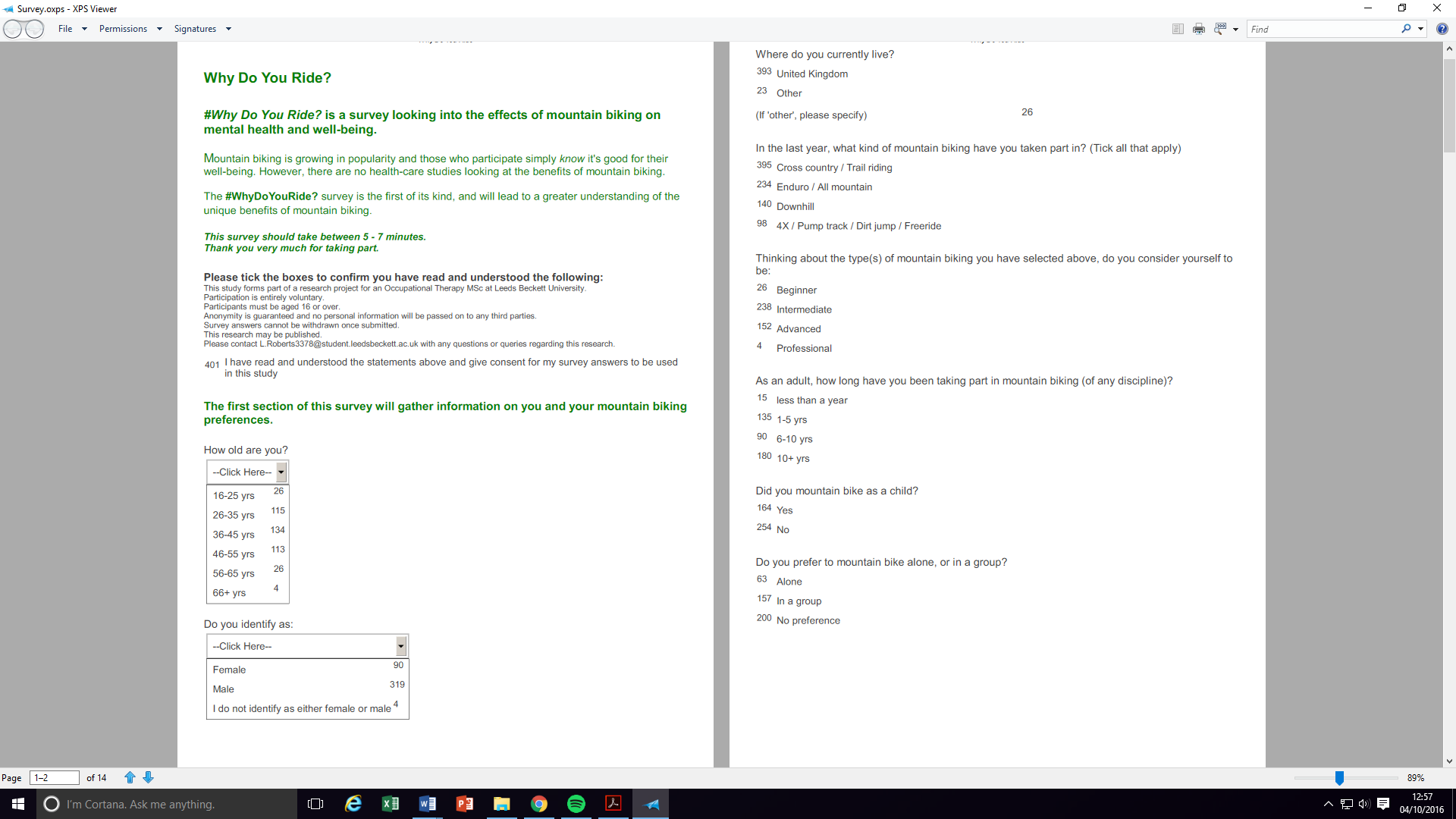


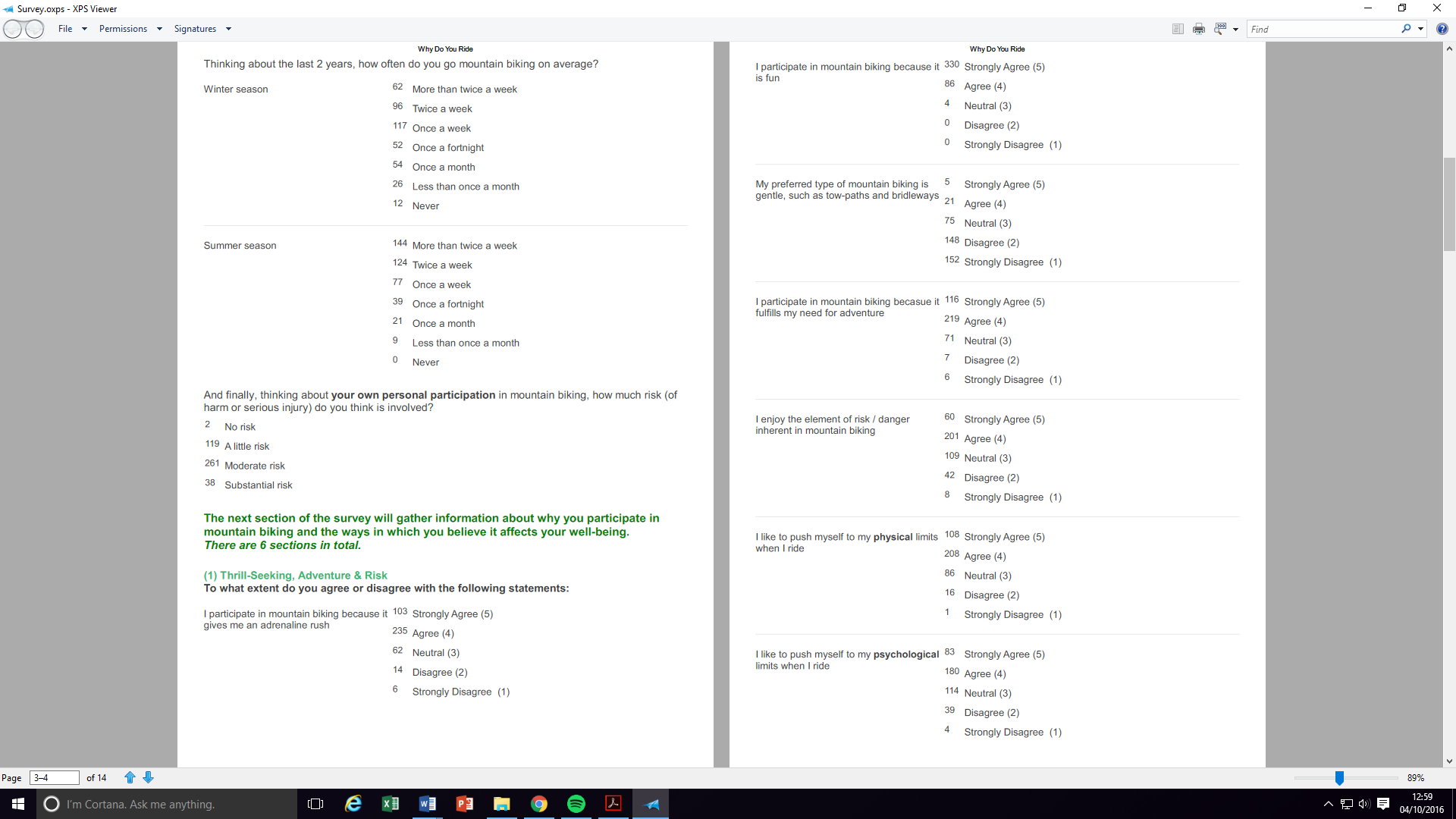


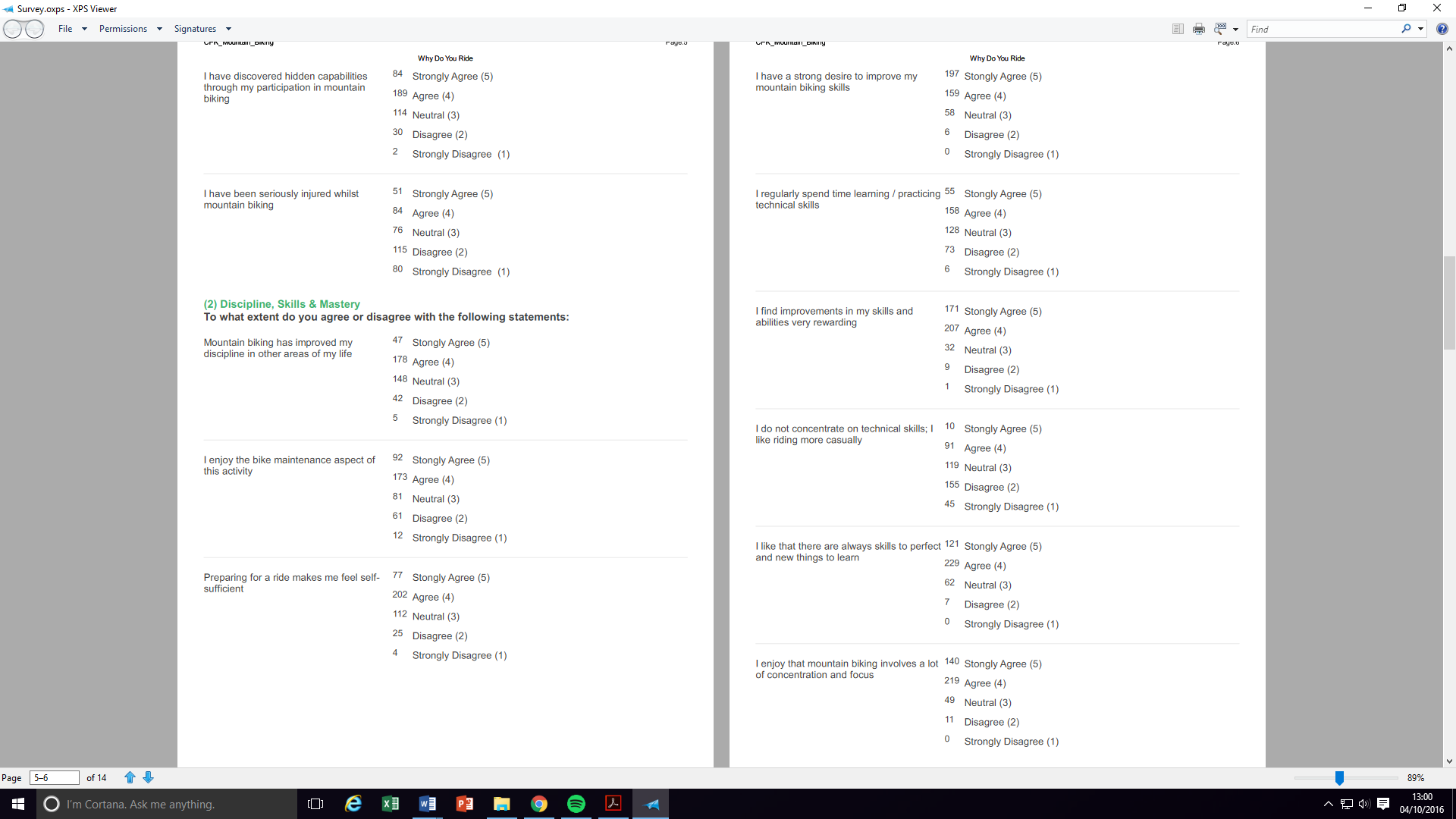


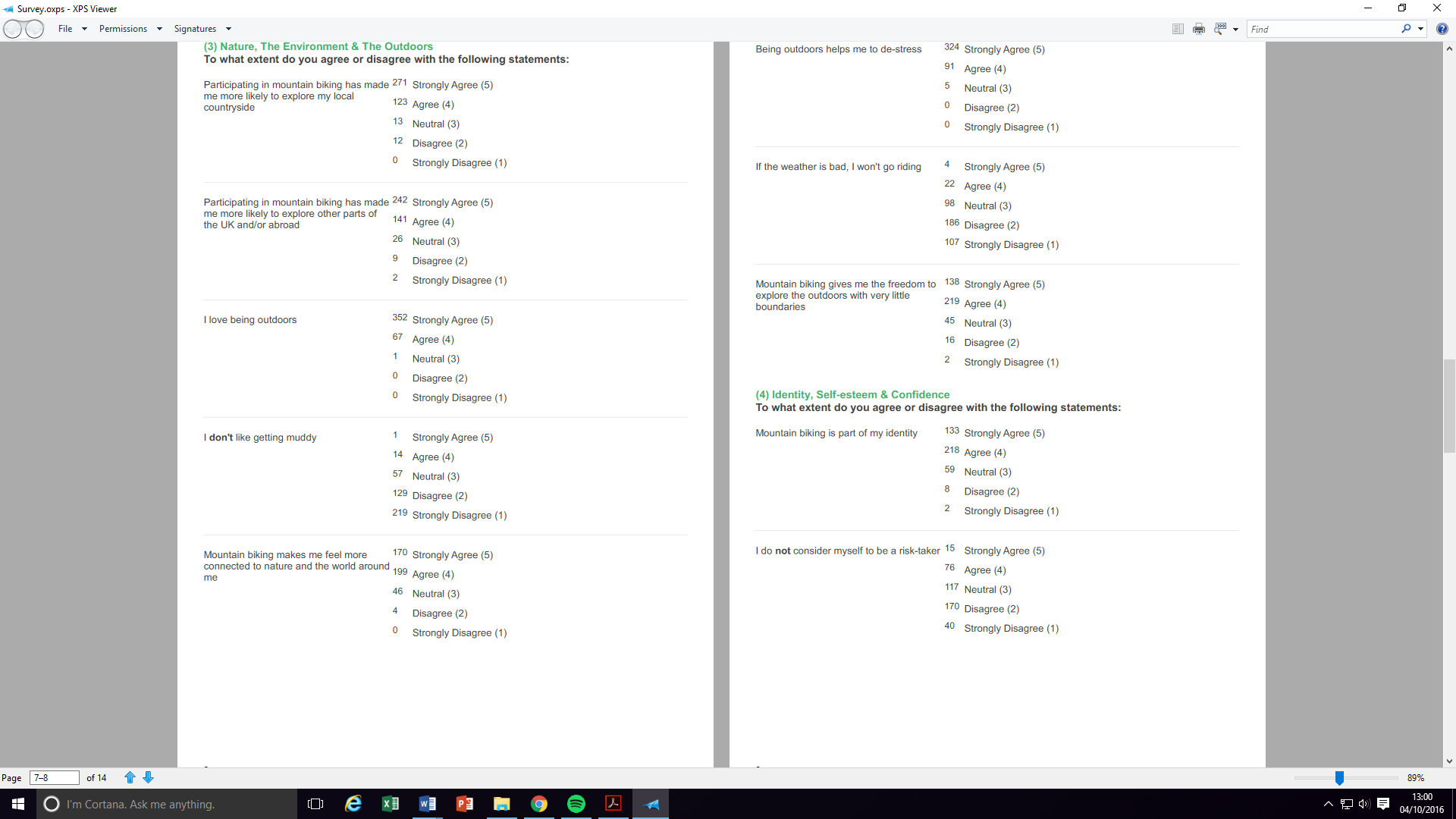


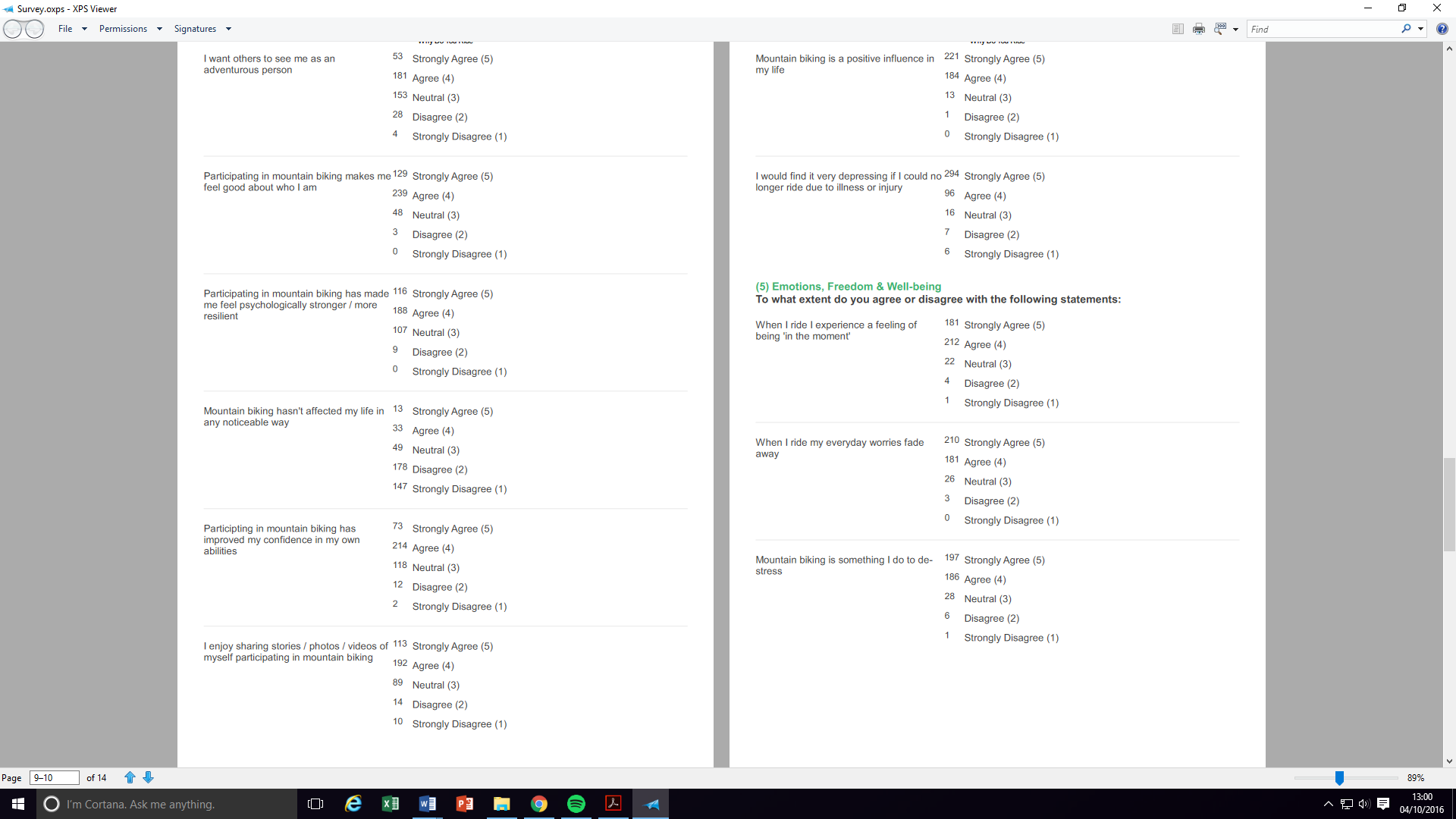


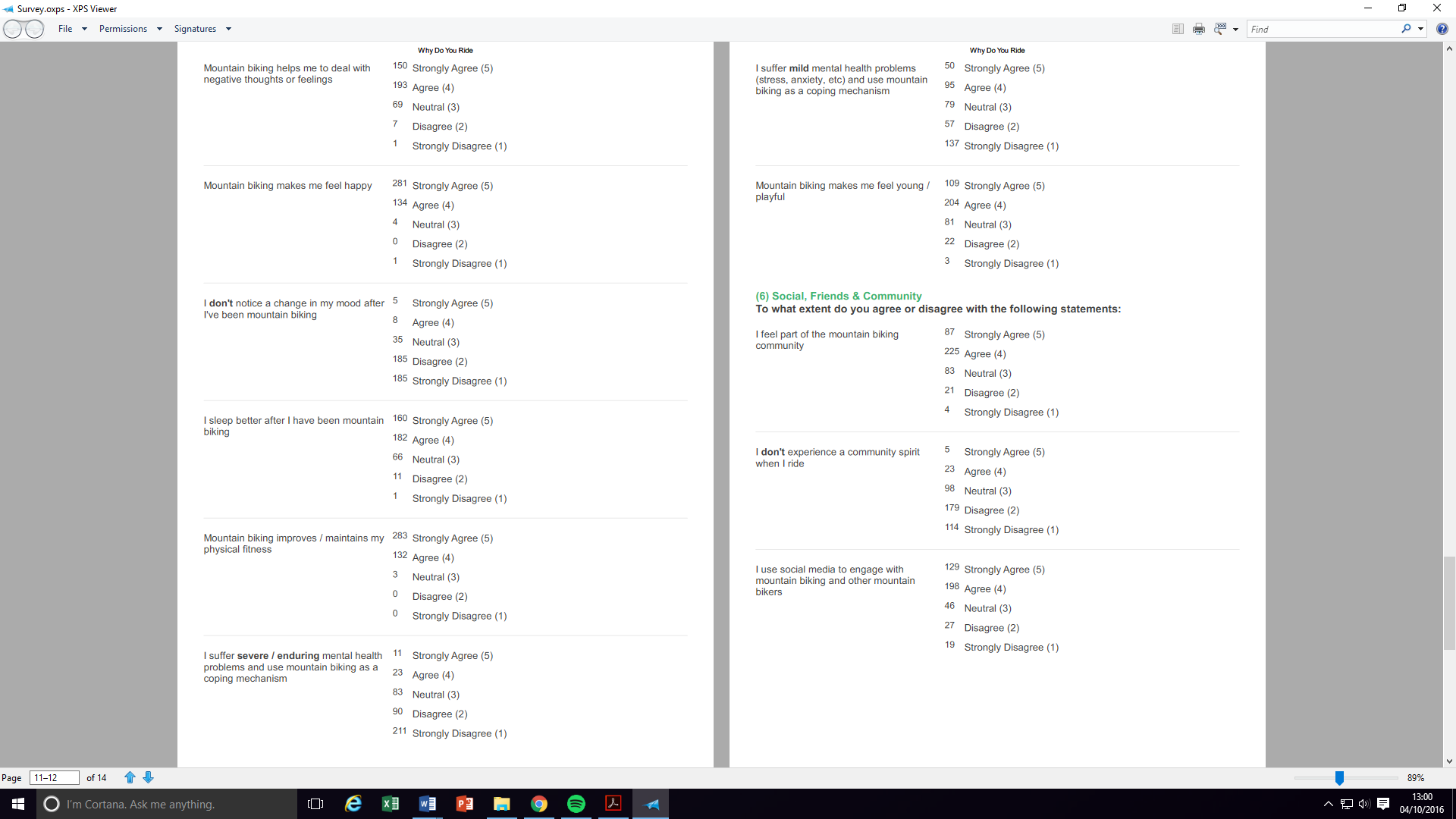


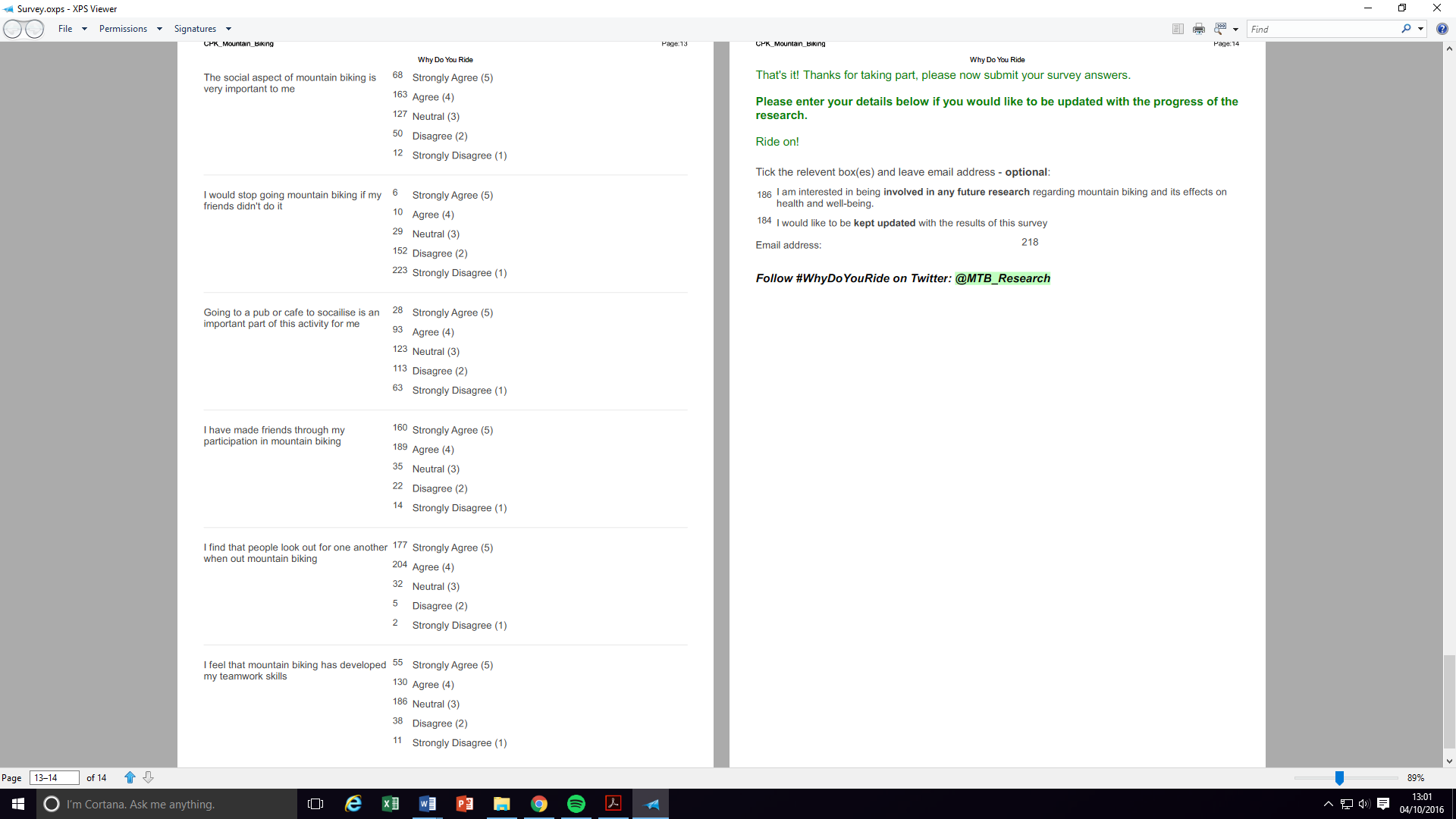


**Survey data collection summary**

Proof of survey data collection from both surveys has been reproduced below.


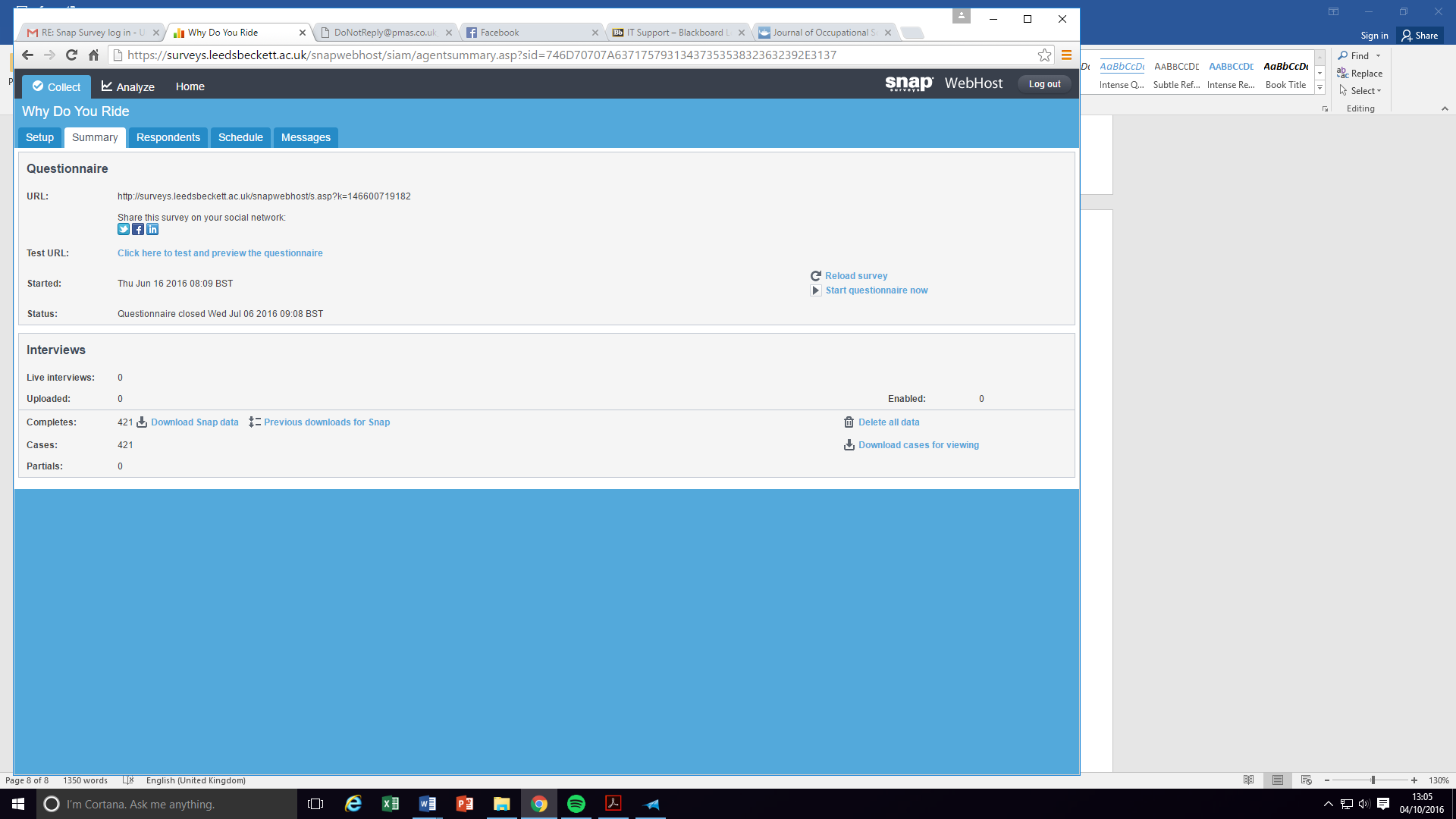

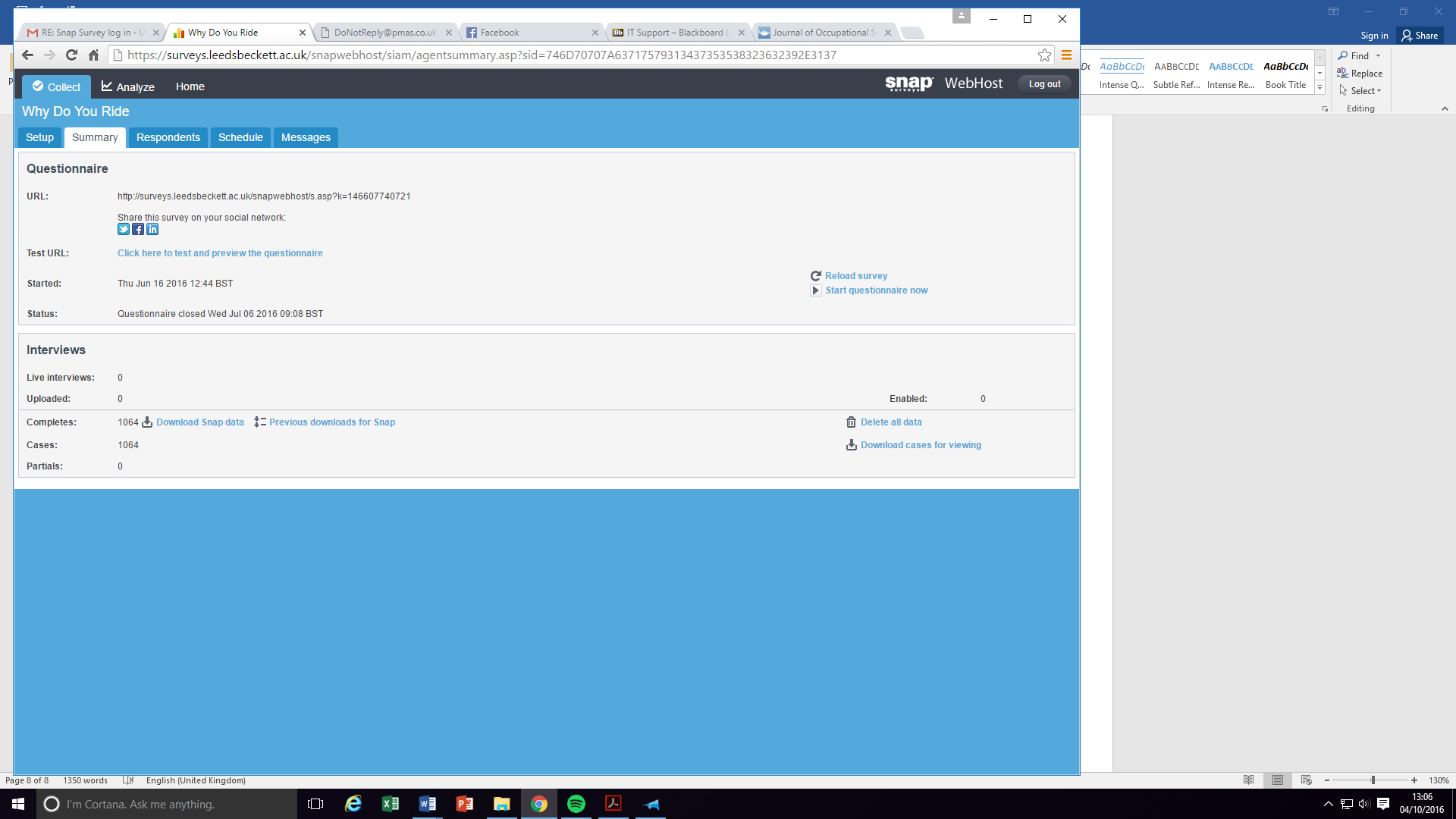

Supplement: Supplementary file 1 [file Table_1.DOCX]
